# Effects of Aquatic Exercise on Balance, Motor Function and Neuromuscular Performance in Children and Young Adults with Disabilities: A Systematic Review

**DOI:** 10.3390/healthcare14152256

**Published:** 2026-07-23

**Authors:** Francisco Javier Cánovas, Paula Redondo-Delgado, Irati Jauregui-Fajardo, Alicja Naczk, Sergio Maroto-Izquierdo

**Affiliations:** 1 i+HeALTH Strategic Research Group, European University Miguel de Cervantes, 47012 Valladolid, Spain; 2 Obra Social del Santuario, Special Education School, 47012 Valladolid, Spain; 3 Department of Physical Education and Sport, Poznan University of Physical Education, 61-871 Poznan, Poland; 4 Department of Health Sciences, Faculty of Health Sciences, European University Miguel de Cervantes, 47012 Valladolid, Spain

**Keywords:** Halliwick, aquatic therapy, aquatic exercise, pediatric rehabilitation, intellectual disability, neuromuscular performance, balance, strength

## Abstract

**Highlights:**

**What are the main findings?**

- Across eight randomized controlled trials involving children and young adults (3–29 years), Halliwick-based aquatic therapy and aquatic exercise consistently improved balance, trunk/postural control, and gross motor function across multiple disability groups.
- Clinically meaningful gains were also observed in functional mobility and neuromuscular performance (e.g., walking capacity, gait speed, and strength measures), with limited but supportive electromyographic evidence suggesting neuromuscular reorganization.

**What are the implications of the main findings?**

- Aquatic interventions, particularly Halliwick-oriented protocols, may be considered task-oriented complements to land-based rehabilitation and appear well tolerated, although safety reporting remains limited to target balance, gait-related function, and strength while minimizing joint loading.
- Future trials should standardize training dose (intensity, water depth, temperature, and progression), incorporate longer follow-up periods, and include direct assessments of muscle structure and architecture (e.g., ultrasound, DXA, MRI) to better distinguish structural from neural adaptations and strengthen clinical guidelines.

**Abstract:**

**Background/Objectives**: Aquatic therapy is widely used in pediatric rehabilitation because buoyancy and water resistance facilitate movement practice under reduced gravitational load. The Halliwick Method is a prominent approach; however, its effects on balance, motor function, and neuromuscular performance in children and young adults with disabilities have not been comprehensively synthesized. This systematic review summarized the effects of Halliwick aquatic therapy and aquatic exercise on balance, strength, and functional mobility in this population. **Methods**: This review followed Cochrane and PRISMA 2020 guidelines and was registered in PROSPERO (CRD42025635215). PubMed, MEDLINE Complete, PEDro, Web of Science, and Google Scholar were searched from inception to November 2025. Randomized controlled trials (≥2 weeks; ≥10 participants per trial) involving individuals aged 3–29 years with disabilities were included. Risk of bias was assessed using the Cochrane RoB2 tool. **Results**: Eight randomized controlled trials (involving 222 participants) met inclusion criteria. Despite heterogeneity in diagnoses and protocols (Halliwick-based hydrotherapy, swimming, strengthening, task-oriented balance training), most studies reported improvements in balance and postural control (BBS/PBS, WOTA2), gross motor function (GMFM), and functional mobility (6MWT, TUG, gait indices). Several trials demonstrated gains in strength, and one reported favorable changes in lower-limb electromyographic activation. Methodological limitations included small samples, limited blinding, variable dosing, and scarce follow-up. Due to heterogeneity in interventions and outcomes, findings were synthesized narratively. **Conclusions**: Halliwick aquatic therapy and aquatic exercise appear to improve balance, motor function, and neuromuscular performance in children and young adults with disabilities. However, evidence is limited by heterogeneity and methodological constraints. More robust, standardized RCTs with direct muscle and neuromuscular assessments are needed.

## 1. Introduction

Aquatic therapy is a recognized approach for promoting mobility in children with disabilities [1]. The properties of water facilitate movement and reduce stress on joints, making it ideal for therapeutic interventions [2]. The Halliwick Method (HM), which focuses on postural control and movement without aids, has been extensively studied in aquatic rehabilitation [1,2].

HM is based on hydrodynamic principles and enables low-impact activities [3]. The buoyancy reduces gravity and facilitates exercises that are impossible on land [3]. Studies have shown that aquatic therapy, including HM, reduces anxiety and improves mood in people with disabilities [4]. It also has a positive effect on cognition in neurological disorders and promotes muscle strength and gait in cerebral palsy [5].

HM is frequently used in children with cerebral palsy [6], autism spectrum disorder (ASD) and other neuromotor disorders. Interventions in children (<14 years) have led to improvements in lower limb musculature [7]. Electromyography studies showed increases on muscle activation and improved coordination after aquatic therapy [8,9]. Indeed, a recent systematic review complement these findings and suggests that aquatic therapy promotes neuromuscular changes that improve locomotion in children with spasticity [10]. In addition, HM has a positive effect on aquatic skills in children with ASD [11]. The available evidence is promising, although further high-quality trials are needed before stronger practice recommendations can be made.

Muscle strength and muscle mass are critical for the development of motor skills in children with disabilities without exacerbating spasticity and are positively associated with autonomy and quality of life [12,13]. Although previous studies and meta-analyses have demonstrated the benefits of aquatic therapy, including the Halliwick Method, on motor function [14], muscle strength [15], and postural stability [16] in children with disabilities, while some research has addressed changes in muscle mass and activation patterns [17], there is a lack of comprehensive synthesis regarding how HM influences both structural and functional outcomes in this population. Despite the limited number of available trials and the heterogeneity of interventions and outcome measures, a systematic synthesis of the current evidence remains necessary. Existing studies are distributed across different diagnostic groups and employ diverse methodological approaches, making it difficult to establish an overall understanding of the potential effects of aquatic interventions in this population. Therefore, a systematic review may help identify current evidence, methodological limitations, and priorities for future research. This gap in the literature limits the ability to establish evidence-based guidelines for the application of HM in pediatric rehabilitation. Therefore, the aim of this systematic review was to synthesize the available randomized controlled trials investigating the effects of Halliwick-based aquatic therapy on balance, motor function, strength, and neuromuscular performance in children and young adults with disabilities.

## 2. Materials and Methods

### 2.1. Search Strategy

This systematic review was conducted in accordance with the Cochrane Handbook for Systematic Reviews of Interventions (version 6.0) and the Preferred Reporting Items for Systematic Reviews and Meta-Analyses 2020 (PRISMA) guidelines. The review protocol was registered in PROSPERO (ID: CRD42025635215).

A comprehensive computerized search was performed in the following databases: PubMed, MEDLINE Complete, PEDro, Web of Science and Google Scholar. The search was conducted from the inception of each database until 15 November 2025, with no restrictions on publication date. The search was performed following the PRISMA guidelines. Trial registries including ClinicalTrials.gov and WHO ICTRP were searched on 18 November 2025. Manual reference searches were conducted on 20 November 2025. The search strategy was developed to address the lack of systematic reviews and meta-analyses on the effects of Halliwick aquatic therapy and aquatic exercise on neuromuscular capacity in children with disabilities. Only articles published in English or Spanish were considered. The search combined keywords and MeSH terms such as “Halliwick”, “aquatic therapy”, “Halliwick aquatic therapy”, “muscle mass”, “children with disabilities”, “children with special needs”, “physical exercise”, “aquatic exercise”, “strength”, using Boolean operators (AND, OR) to optimize sensitivity and specificity. For Google Scholar, the first 200 results sorted by relevance were screened, as recommended for systematic reviews, to improve reproducibility. The search was conducted using predefined keywords and Boolean combinations. The reference list of all selected studies, as well as relevant reviews, conference proceedings, and abstracts, were manually screened to identify additional studies not captured by the electronic search. Please see Supplementary File S1.

### 2.2. Study Selection

References to selected publications included original articles, abstracts, and conference proceedings. To identify relevant articles, the titles and abstracts of all publications selected after the initial search were analyzed, looking for studies that had carried out an aquatic therapy or Halliwick therapy program, as well as examining changes in musculature, including neuromuscular capacity. Thus, in addition to the titles identified in the initial search, the titles and abstracts of all newly identified publications that included interventions based on aquatic therapy or Halliwick therapy for children and adolescents with disabilities were analysed. Studies whose titles and abstracts did not include references to the use of any aquatic therapy or Halliwick therapy program for children and adolescents with disabilities or that did not meet other eligibility criteria, such as study design or population, were excluded. Full articles were retrieved if the abstract did not provide sufficient information to establish eligibility or if the article abstract had passed the initial eligibility review.

Titles and abstracts identified through the initial search were independently screened by two reviewers (JC-H and SM-I) to assess eligibility. Disagreements were resolved through discussion. Duplicate records were removed using an online deduplication tool (https://tera-tools.com (accessed on 14 July 2026)) and manual verification. The systematic review software Rayyan (https://www.rayyan.ai (accessed on 14 July 2026)) was used to facilitate blinded screening and minimize bias.

Population: Children and young adults with disabilities aged between 3 and 29 years, representing the lower and upper age limits of the eligibility criteria.

Intervention: Intervention and physical exercise in the aquatic environment and Halliwick therapy.

Comparison: Children and young adults with disabilities receiving usual care, no intervention, minimal physical activity, conventional rehabilitation, or alternative exercise-based interventions.

Outcome:

Primary outcomes: Balance; motor function; functional mobility; neuromuscular performance.

Secondary outcomes: Strength; functional capacity; quality of life; mental health outcomes; metabolic health

Context: Aquatic therapy or Halliwick therapy programs for children or adolescents with disabilities.

### 2.3. Eligibility Criteria

Studies were included if they met the following criteria:

1. Randomized controlled trials (RCT) published in peer-reviewed journals.
2. Studies examining effects between 2 and 40 weeks of Halliwick aquatic therapy or aquatic exercise on strength or balance in children with disabilities. The duration of interventions ranged from 2 to 40 weeks based on the characteristics of the included studies and was not used as an eligibility restriction during the search process.
3. Studies providing quantitative data on changes in strength or balance or other relevant structural parameters.
4. Assessment of neuromuscular function through any isometric, isokinetic or isotonic maximum strength test with or without complementary surface electromyography (EMG) assessment.
5. Studies published in English or Spanish.

The following studies were excluded:

1. Non-randomized studies.
2. Age range out of interest.
3. Studies without an intervention or with intervention lower than 2 weeks.
4. Studies without quantifiable data on strength or balance.
5. Studies not involving aquatic therapy or the Halliwick Method.
6. Studies with a total sample size of fewer than 10 participants per trial were excluded; the sample size criterion was applied to the overall study population and not to individual study groups.
7. Letters to the editor, commentaries, conference abstracts, or other non-original research.

### 2.4. Data Extraction

Data extraction was performed independently and in duplicate by two authors (JC-H and SM-I) using a standardized data extraction form. Discrepancies were resolved through discussion. The following data were extracted from each included study: Study identification information (authors, year of publication, journal), study design (RCT), sample size, participants’ characteristics (age, sex, disability type), intervention characteristics (type of aquatic therapy, duration, frequency, intensity), neuromuscular capacity assessment methods, outcome measures (balance, strength, lean mass, etc.), and neuromuscular results (e.g., muscle strength, torque, EMG). Means, standard deviations, *p*-values and effect sizes were recorded for each outcome. When necessary, authors were contacted to obtain missing data.

### 2.5. Data Synthesis and Analysis

A descriptive analysis of the included studies was conducted, summarizing study characteristics, participant demographics, intervention details, and outcome measures. Due to heterogeneity in populations, interventions, and outcome measures, a quantitative meta-analysis was not feasible. Therefore, a narrative synthesis was conducted to summarize and compare findings across studies. Studies were synthesized according to predefined primary and secondary outcome domains, allowing a structured narrative interpretation of intervention effects across outcome categories.

### 2.6. Assessment of Methodological Quality and Risk of Bias

The methodological quality and risk of bias of included studies were independently assessed by two reviewers (JC-H and SM-I) using the Cochrane Risk of Bias 2 (RoB2) tool for RCTs. Disagreements were resolved through discussion. The RoB2 tool assesses bias arising from the randomization process, deviations from intended interventions, missing outcome data, measurement of the outcome, and selection of reported results. Studies were classified as having low risk of bias, some concerns, or high risk of bias. Risk-of-bias figures were created using Robvis software (https://mcguinlu.shinyapps.io/robvis/ (accessed on 14 July 2026)). The overall certainty of evidence was assessed using the Grading of Recommendations Assessment, Development and Evaluation (GRADE) approach, considering risk of bias, inconsistency, indirectness, imprecision, and publication bias.

## 3. Results

### 3.1. Study Selection

**Figure 1:**
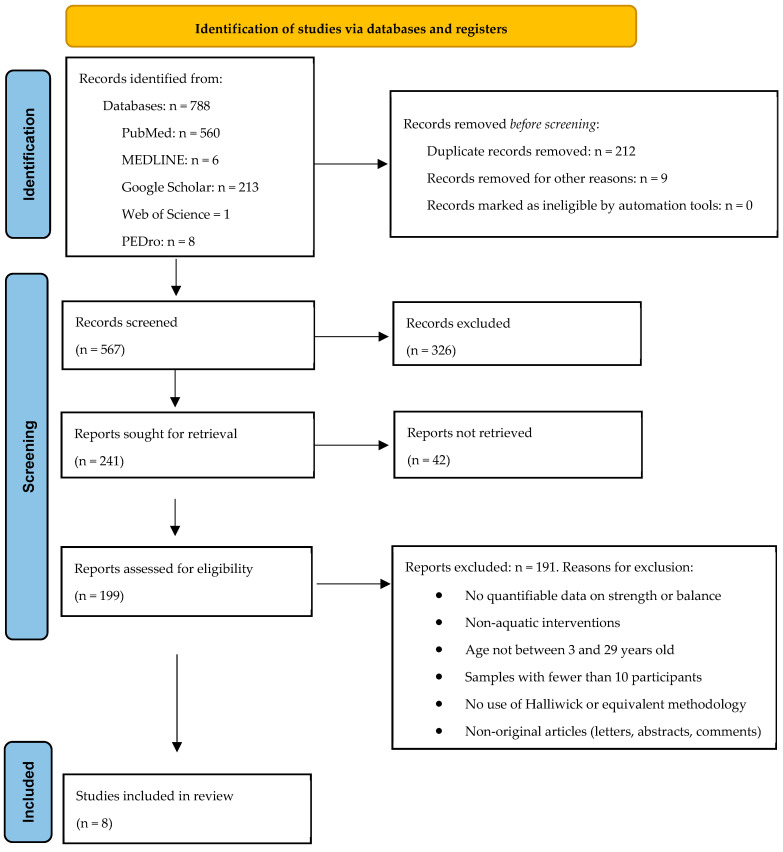
PRISMA flow chart for study selection.

A total of 788 studies were identified through the initial search in PubMed, MEDLINE Complete, PEDro, Web Of Science and Google Scholar (Figure 1). After removing duplicates using an online deduplication tool and manual selection, a total of 567 articles remained for review. Of these, 326 were excluded based on their title, and of these, 42 were excluded because they could not be found, 199 were read in abstract and full text, and 191 were excluded because they did not involve intervention in children and young adults with disabilities (*n* = 15), no quantifiable data on muscle mass, strength or balance (*n* = 13), non-aquatic interventions (Halliwick or equivalent methodology) (*n* = 7), participants’ age did not range between 3 and 29 years old (*n* = 141), samples with less than 10 participants (*n* = 6), no RCT (*n* = 8) and different language from English or Spanish (*n* = 1). Finally, 8 studies met all the inclusion criteria and were included in the qualitative synthesis. A complete list of excluded references and the reasons for their exclusion can be found in Supplementary File S2.

### 3.2. Included Studies Characteristics

The studies included in this review were primarily randomized controlled trials (RCTs) involving paediatric and young adult populations diagnosed with various conditions. The eight included studies collectively enrolled 222 participants. Participants presented a range of disabilities, including cerebral palsy, autism spectrum disorder, and other neurodevelopmental conditions. Most interventions were based on the Halliwick Method or Halliwick-oriented aquatic exercise programs, with intervention durations ranging from 2 to 40 weeks. Participant ages ranged from 3 to 29 years

Following review of the content, the characteristics of the interventions are shown in the following table (Table 1), including *n*, duration, days and implementation, as well as the population and diagnosis:

### 3.3. Outcomes Analysed and Main Results

The set of studies reviewed presents converging evidence regarding improvements in motor skills, balance, postural control, and quality of life as the main outcomes derived from aquatic interventions or structured physical programmes in populations with different types of neuromotor or intellectual disabilities. Despite methodological and population heterogeneity, there is conceptual consistency in the selection of functional variables as indicators of therapeutic efficacy.

Gross motor function is one of the most recurrent outcomes in the studies analysed. Two studies used the Gross Motor Function Measure (GMFM) to assess overall mobility in children with cerebral palsy (CP) [14,20]. In both cases, significant improvements were observed in post-intervention scores, especially in the group that followed programmes based on the Halliwick concept. However, while sustained improvement was reported during the three months of intervention [20], partial deterioration was also observed at follow-up, suggesting that therapeutic continuity is a determining factor in maintaining functional benefits [14].

At the same time, a significant increase in gross motor skills (measured with GMFM and FIM) in children with CP following swimming and aquatic strengthening programmes has been confirmed, results that coincide with previous findings on the effectiveness of aquatic programmes in optimising motor control [21].

Likewise, including complementary measures such as the Six-Minute Walk Test (6MWT), walking speed and the Timed Up and Go (TUG), increases in functional mobility and walking efficiency were evident after aquatic training focused on balance [19]. Taken together, these results underscore that aquatic programmes promote global and functionally relevant improvements in gross motor skills, with transfer to daily life.

Balance emerges as a cross-cutting and central outcome in most studies. In the terrestrial setting, there is agreement that both static and dynamic balance are indicators of therapeutic efficacy, using instruments such as the Modified Stork Test, the Heel-to-Toe Walking Test, the Paediatric Balance Scale (PBS) and the Dynamic Gait Index (DGI). They report statistically significant improvements in postural control, highlighting the benefits of both karate kata training and aquatic training, demonstrating the functional equivalence of both modalities in enhancing body stability [18,19].

In aquatic contexts, the effectiveness of Halliwick therapy in improving balance is reaffirmed, evidenced by significant increases in the Berg Balance Scale (BBS) and in the balance subscales of the Water Orientation Test Alyn 2 (WOTA 2) [14,20]. This is capable of maintaining post-intervention improvements, in contrast to the GMFM results, complement the idea that the effects on balance are more sustainable than those on gross motor skills [14].

More recent studies also show substantial improvements in balance in children with intellectual disabilities and CP [21,24], respectively, while the aquatic environment promotes neuromuscular reorganisation and postural control even in the absence of visual feedback, showing increases in electromyographic activation and motor coordination [23]. All these studies complement the hypothesis that the aquatic environment promotes sensory integration, trunk control and postural stability, which are essential factors for improving dynamic balance and motor autonomy.

Trunk control was assessed using the Trunk Control Measurement Scale (TCMS), with significant improvements found after the intervention [19]. Indirectly, this points to a strengthening of the core and axial muscles, suggesting that trunk control is a cross-cutting element underlying the improvement in balance and overall motor function in all the interventions analysed [21,22].

Outcomes related to gait and functional mobility incorporated measures of functional displacement (6MWT), walking speed, energy expenditure (EEI), and risk of falling (TUG). The results showed clinically relevant increases in distance walked and walking speed, with high effect sizes (d = 1.09), demonstrating a significant improvement in locomotor efficiency [19].

Improvements in dynamic balance are indirect predictors of mobility and functional autonomy [18]. At the same time, they confirmed similar advances in functional capacity and independence in mobility tasks, complement the direct relationship between postural control, balance and gait efficiency [21].

The psychosocial and quality of life dimension was addressed using the Child Health Questionnaire—Parent Form 50 (CHQ-PF50) and the Visual Analog Scale (VAS), recording significant improvements in parental perception of well-being and gait quality [19]. The theoretical discussion links improvements in balance with greater social and communicative functioning in children with ASD [18]. Similarly, regular aquatic practice promotes autonomy, social integration, and positive self-perception of motor competence in people with intellectual disabilities [24].

Overall, the studies reviewed support the claim that structured aquatic and motor interventions produce consistent improvements in motor function, balance, trunk control, muscle strength, aerobic capacity, and quality of life, with measurable effects in both objective and subjective domains. The functional transfer of balance to daily life was emphasised [18,19], demonstrating the sustained efficacy of Halliwick hydrotherapy in CP [14,20], providing evidence on neuromuscular plasticity [21,23], while extending the evidence to populations with intellectual or sensory disabilities, confirming the benefits of aquatic exercise for overall health and functional participation [22,24]. All of this supports the view that aquatic interventions are increasingly recognized as multidimensional and evidence-informed strategies that promote improved motor, physiological, and psychosocial outcomes in populations with special needs, although evidence regarding safety reporting remains limited, reaffirming their value within rehabilitation and adapted physical activity programmes.

### 3.4. Risk of Bias and Methodological Characteristics

**Figure 2:**
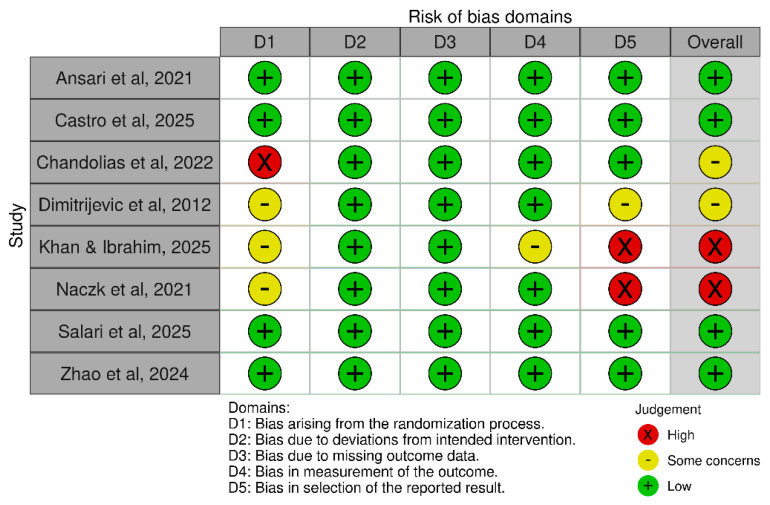

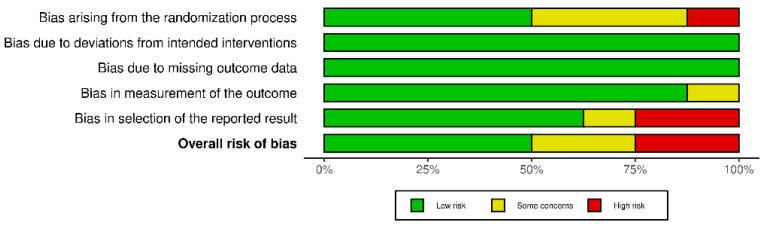
Overall risk of bias of articles included in a systematic review [18,19,20,21,22,23,24].

The risk of bias was assessed using the Cochrane Risk of Bias 2 (RoB2) tool for randomized studies. The proportion of studies identifying bias or more design elements is presented in Figure 2, and The RoB2 scores of the included studies are presented in Supplementary File S3. Concerns about underlying bias were mainly due to issues arising from the randomization process and deviations from intended interventions, such as a lack of clarity regarding blinding (e.g., whether participants and investigators were aware of their assigned intervention during the trial).

## 4. Discussion

This systematic review suggests that aquatic exercise consistently improves balance and postural control in children and adolescents with disabilities, including autism spectrum disorder, cerebral palsy, intellectual disability, Down syndrome, and visual impairment [14,18,19,20,21,22,23,24]. In ASD, improvements in static and dynamic balance are frequently reported [18], while in cerebral palsy, aquatic programmes, particularly Halliwick-based and trunk control protocols, demonstrate gains in postural control and global motor function [14,19,20], with data from other additional studies not included in the review due to exclusion criteria provide contextual support for potential mechanisms, although the conclusions of this review are based exclusively on the included studies [10,25,26]. These effects are strongly linked to the properties of the aquatic environment, such as buoyancy, viscosity, resistance, and hydrostatic pressure, which reduce gravitational load and require continuous postural adjustments, thereby enhancing proprioception and motor safety [18,19,20,23]. Continuous disturbances in water promote agonist-antagonist coactivation and neural plasticity according to studies not included in the review due to exclusion criteria [9,27], which explains the rapid improvements in balance, postural efficiency, and motor control [18]. In related studies not included in this review, multisensory stimulation further promotes somatosensory and vestibular integration [28], which contributes, along with the reviewed articles, to improving orientation, reducing fear of movement, and increasing adherence and participation [22,26,28].

Improvements in balance and postural control achieved through aquatic exercise translate into improved gait and functional mobility, which is reflected in better performance in walking ability and activities of daily living, including outcomes such as the 6MWT, GMFM, and PBS [18,19,24]. The aquatic environment allows children to practise complex motor patterns and gait-related tasks that may be inaccessible on land, facilitating safer movement with less pain, fear and fatigue [21] complementing this interpretation with external literature used only for contextual discussion in the review due to exclusion criteria [29]. This promotes the transfer of benefits to walking on land and daily activities, especially in children with cerebral palsy and other neurodevelopmental disorders [21]. Other reviews of aquatic interventions report positive effects on mobility and quality of life, while highlighting the need for greater standardisation of protocols [15]. Furthermore, the psychosocial benefits of the aquatic environment, such as increased motivation, emotional security, sensory regulation, and social interaction, further improve functional participation and mobility outcomes [30], also appearing in other articles not included in the review [31,32]. References to studies outside the review are presented only as contextual background and are not part of the evidentiary synthesis supporting the review conclusions.

Although the studies reviewed do not directly assess muscle mass using DXA, US or MRI, they systematically document improvements in strength, power, neuromuscular activation and motor control, allowing us to infer functional and neural adaptations rather than purely structural hypertrophy. Increases in strength and power have been reported using dynamometry, functional tests, and GMFM results [19,21,22], including 15–30% increases in lower limb strength [22,24], as well as improvements in hand strength, power, and muscle endurance following structured swimming protocols, documented alongside publications not included in the review [21,25]. Neuromuscular adaptations are supported by EMG results showing increased activation, higher MVIC percentages and reduced activation latencies [23], consistent with the central adaptations described in the literature not included in the review on the V wave and H reflex [33]. The buoyancy of water allows for a wide range of joint movement and prolonged mechanical loading, which may contribute to improved functional muscle performance, while enabling high training volumes with low joint stress and reduced peripheral fatigue, also referenced in articles not included in the review [21,22,34]. In related work not included in the present review, the uniform resistance provided by water is comparable to elastic resistance training [35], and rapid concentric-eccentric muscle contractions can improve power and speed of force development [36]. These mechanisms align with paediatric strength models on land that show increases in strength without exacerbating spasticity in children with cerebral palsy [37]. Water training also optimises movement patterns, reduces pathological co-activation, and improves postural control [10,19], supporting the interpretation that aquatic exercise may enhance functional strength relevant to activities of daily living, as well as meaningful and robust neuromuscular adaptations comparable to those observed with low- or moderate-intensity strength training.

The included studies have significant methodological limitations that affect the robustness and generalisability of the results, including small sample sizes and low representativeness of the samples [18,19,20], diagnostic and functional heterogeneity (different GMFCS levels, wide age variability, and low inclusion of girls), the diversity of aquatic protocols used (Halliwick, swimming, Ai Chi, strengthening, water play), as well as variability in key parameters such as temperature, depth, duration, and intensity of the sessions [19,21].

Added to this is the inherent difficulty of blinding therapists and participants, the absence of three-dimensional kinematic analysis in cerebral palsy [19], the limited quantification of mechanical load and internal and external load [21], and the almost total absence of direct measurements of muscle mass and architecture (ultrasound, DXA or MRI), with functional results being used in most cases, with a single exception that directly incorporates electromyography [23].

The studies reviewed report null or non-significant results in key variables, especially in control groups, in intergroup comparisons, or in certain functional domains, which limits the consistency of the available evidence [14,18,19,20,21,22,23,24]. In this regard, several studies show no significant changes in static and dynamic balance, as well as in other functional abilities, despite improvements in strength, aquatic skills, or overall motor performance in different tests such as Eurofit [18,22]. Likewise, no relevant differences between intervention groups are described in certain scales of balance, quality of life, or functional classification, even when clinically relevant advances are recorded in other outcomes [19,21,24]. Similarly, control groups undergoing conventional physiotherapy or no intervention did not show significant improvements in most studies [14,18,20], and in the few studies with follow-up, a partial attenuation of functional benefits is observed after completion of the programme [14]. Finally, neuromuscular results show heterogeneous responses, with non-uniform improvements between muscles or activation patterns, provide mechanistic context the variability of the observed effects [23]. Taken together, this repeated occurrence of non-significant results, coupled with the generalised absence of longitudinal follow-ups, reduces the capacity for extrapolation and highlights the need for more robust and comparable designs [19,20]. No serious adverse events were reported in the included studies; however, reporting of harms was limited and safety conclusions should be interpreted cautiously.

Based on these limitations, future lines of research are proposed aimed at improving methodological quality, including the systematic incorporation of direct measurements of hypertrophy and muscle architecture (quantitative ultrasound, DXA, MRI; cross-sectional area, pennation angle, fascicular thickness and length), anabolic biomarkers related to pathways such as mTOR, and advanced neuromuscular assessment tools such as high-density EMG, H-reflexes and V-waves located in other articles not ultimately included in the review [33], force platforms, accelerometry and submersible inertial sensors, as well as neuroimaging and EEG studies to explore cortical plasticity associated with aquatic training. It is also a priority to evaluate functional transfer to activities of daily living, social participation according to the ICF, the permanence of improvements, and possible cognitive and neuroplastic effects [19,38], rigorously comparing aquatic programmes with land-based strength training or hybrid protocols controlling volume and intensity [39], analysing dose–response relationships (frequency, duration, depth), developing standardised protocols according to cerebral palsy subtypes [16,25], studying the interaction between sensory deficits and motor adaptations, and moving towards multicentre randomised clinical trials with adequate sample sizes to compare the differential effectiveness of modalities such as swimming, strengthening, Ai Chi or aquatic HIIT [40]. Multiple outcomes were assessed without multiplicity adjustment, which may increase the risk of type I error and should be considered when interpreting the findings.

From a practical perspective, the findings of this review support the systematic inclusion of aquatic exercise and Halliwick-based interventions as part of rehabilitation and adapted physical activity programs for children and young adults with disabilities. The consistent improvements observed in balance, postural control, gait efficiency, muscle strength, and functional mobility suggest that aquatic environments provide a safe and effective setting to train motor skills that are often limited on land due to spasticity, poor balance, or fear of falling [14,18,20]. Halliwick-based protocols, in particular, appear especially suitable for improving postural control and functional balance in children with cerebral palsy and intellectual disabilities, facilitating transfer to walking performance and daily activities [19,24]. In addition, swimming-based and aquatic strengthening programs can be used to enhance muscle power, endurance, and functional independence without exacerbating spasticity, making them appropriate for children with spastic cerebral palsy and Down syndrome [21,22]. The documented neuromuscular adaptations, including increased muscle activation and reduced onset times, indicate that aquatic exercise may also be a valuable tool for promoting neuromuscular reorganization in populations with sensory or visual impairments [23]. Taken together, these results suggest that clinicians, physiotherapists, and adapted physical activity professionals can tailor aquatic programs, adjusting depth, task complexity, and movement goals, to target balance, strength, gait, or psychosocial participation, thereby integrating aquatic therapy as a complementary or alternative intervention within multidisciplinary rehabilitation pathways for children and young adults with disabilities.

The available evidence shows that aquatic exercise is a potentially beneficial interventions for improving mobility, postural control, and motor function in children with neurological disabilities. Although the studies do not directly measure muscle mass or muscle architecture, their functional findings, together with supportive evidence from the broader literature, suggest the possibility of relevant neuromuscular adaptations and potential structural adaptations that require confirmation through direct assessment methods. The development of more robust research will allow for a clearer definition of the potential of the aquatic environment as a stimulus for improving strength and clarifying the potential role of hypertrophic adaptations in pediatric populations with disabilities.

This systematic review presents several limitations. First, the number of included studies was small, with heterogeneous populations and intervention protocols. Second, methodological limitations such as lack of blinding, small sample sizes, and limited follow-up periods reduce the generalizability of findings. Third, few studies directly assessed neuromuscular adaptations using objective measures such as imaging or electromyography. These limitations highlight the need for more robust randomized controlled trials with standardized protocols. Consequently, the overall certainty of the available evidence remains limited and findings should be interpreted with caution.

## 5. Conclusions

This systematic review suggests that Halliwick-based aquatic therapy and structured aquatic exercise programs may provide beneficial effects on balance, postural control and gross motor function in children and young adults with disabilities. However, given methodological heterogeneity, limited certainty of evidence and scarce harms reporting, these findings should be interpreted cautiously. The synthesized evidence indicates that the unique hydrodynamic properties of the aquatic environment facilitate task-oriented motor learning and functional mobility gains that are often restricted in land-based settings; however, the high degree of methodological heterogeneity regarding intervention protocols, diagnostic groups, and dosing parameters underscores the need for caution in generalizing these results. While functional and neuromuscular benefits appear consistent, further high-quality trials incorporating objective assessments of muscle architecture are required to clarify the extent of structural adaptation.

**Figure 3:**
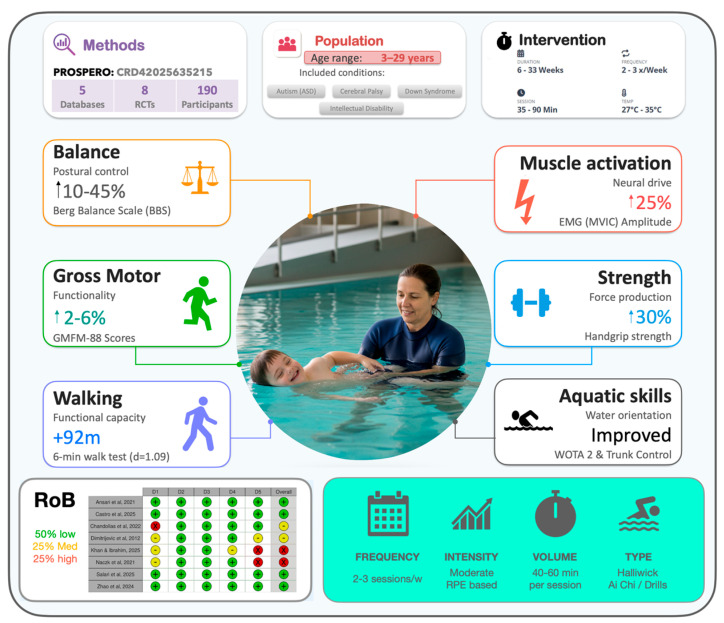
Schematic overview of the main findings across included studies. Numerical values reflect results from individual studies or observed ranges and should not be interpreted as pooled effect estimates or meta-analytic results [18,19,20,21,22,23,24].

In conclusion, aquatic interventions represent valuable multidimensional tools within multidisciplinary rehabilitation pathways. Future research should prioritize large-scale, multicenter randomized controlled trials with standardized protocols and objective neuromuscular assessments to establish evidence-based guidelines and clarify the long-term sustainability of these functional benefits in pediatric and young adult populations with special needs (Figure 3).

## Figures and Tables

**Table 1:** Included studies characteristics.

Study and Country	Sample and Study Characteristics	Aquatic Intervention Characteristics	Outcomes and Main Findings *
**[18]** **Iran**	30 children (age: 8–14; diagnosed with ASD) **Experimental group A (*n* = 10):** aquatic training. **Experimental group B (*n* = 10):** kata techniques (martial arts). **Control group (*n* = 10):** no specialised intervention. Pre-test and post-test (before and after the 10-week intervention).	**Duration:** 10 weeks, 60 min/session **Frequency:** 2 sessions/w. **Exercises:** Water-based motor exercises (aquatic movements and swimming-like drills) focused on balance, coordination, and body control in a buoyant environment. **Control group:** Continued usual daily and school activities without structured training. Static balance (Stork Stand Test) and dynamic balance (Walk & Turn Test)	↑↑↑ Stork Stand Test ↑↑↑ Walk & turn test The **control group** showed no significant changes.
**[19]** **Brazil**	16 children (age: 6–8; diagnosed with BSCP) **Balance group (BG)**, *n* = 8. **Trunk group (TG)**, *n* = 8. Assessments were conducted 1 week before and 1 week after the 8-week intervention. Two-arm, parallel, and blinded clinical trial	**Duration:** 8 weeks, 35 min/session **frequency:** 2 sessions/w. **Exercises:** one land-based physical therapy session/week. Stretching hip adductors/flexors, knee flexors; strengthening hip abductors). **BG aquatic protocol:** Exercises emphasised activation of dorsiflexors, knee and hip extensors, balance strategies, lower-limb weight bearing and gait-related tasks (task-oriented, gait-similar activities). **TG aquatic protocol:** Exercises emphasised trunk mobility and stretching (hip flexors), activation of scapular stabilizers, trunk flexors/extensors/rotators, and postural control in standing. **Setting:** Indoor heated pool (33–35 °C). 6-Minute Walk Test (6MWT)—distance (m), Gait speed (GS)—derived from 6MWT (m/s), Trunk Control Measurement Scale (TCMS)., Pediatric Balance Scale (PBS), Timed Up and Go (TUG), Dynamic Gait Index (DGI), Energy Expenditure Index (EEI) (calculated from HR + speed) and Parent-reported (PGQ VAS): Child Health Questionnaire—Parent Form 50 (CHQ-PF50) and Visual Analog Scale for gait (VGAS).	6MWT distance: ↑ BG (+59%)–↑ TG (+18%). Between-group difference: 92.62 m, ↑ favoured BG. GS: ↑ BG (+102%)–↑ TG (+39%). Between-group difference: +0.26 m/s, ↑ favoured BG. TCMS: ↑↑ BG (+54%)–↑ TG (+60%). PBS: ↑ BG (+38%)–↑ TG (+40%). DGI: ↑ BG (+44%)–↑ TG (+69%). TUG: ↑ BG (−57%)–TG (−13%) PGQ VAS: ↑ BG (+45%)–↑ TG (+60%). CHQ-PF50: ↑ BG (+10%)–↑ TG (+12%).
**[20]** **Greece**	16 children (age: 3–16; diagnosed with CP) **Experimental group (*n* = 10):** Halliwick-based hydrotherapy. **Control group (*n* = 6):** Traditional land-based physiotherapy twice weekly. Assessments were conducted before (pre-test) and after (post-test) the 3-month intervention.	**Duration:** 12 weeks, 45 min/session **Frequency:** 2 sessions/w. **Exercises:** Halliwick concept- One-to-one ratio, no flotation aids used. Play-based activities to improve postural control, breathing, balance, and mobility in the water. **Setting:** Indoor heated pool (32–33 °C, 1.20 m depth). Berg Balance Scale (BBS), Gross Motor Function Measure (GMFM) and Base of support/weight distribution: Footprint plate (pressure plate analysis).	↑ GMFM (+2.1%) ↑ BBS (+9.6%) BS/WD (−8.7% RF + 11.3% LF) The **control group** showed no significant changes.
**[14]** **Serbia**	27 children (age: 5–14; diagnosed with CP) **Experimental group (*n* = 14):** Aquatic intervention. **Control group (*n* = 13):** No intervention. Assessments were conducted baseline (pre-test), After 6 weeks (post-test) no therapy during follow-up, After 9 weeks (3-week follow-up) no therapy during follow-up.	**Duration:** 6 weeks, 55 min/session **Frequency:** 2 sessions/w. **Exercises:** One-to-one ratio. Warm-up (10 min): Walking forward/backward, jumping, gentle mobility. Aquatic exercises (40 min): Prone/back gliding and floating, Blowing bubbles, Basic swimming strokes (breaststroke, backstroke, freestyle), Diving to the pool bottom. Play (5 min): Games with balls, chasing, etc. **Setting:** Indoor heated pool (27.7 °C, 0.7–1.8 m depth). Gross Motor Function Measure (GMFM−88): Assessed land-based gross motor function and Water Orientation Test Alyn 2 (WOTA 2): Evaluated aquatic skills based on the Halliwick concept: WMA = Mental adaptation to water, WSBM = Water balance and movement skills, WTOT = Total aquatic skill score.	↑ GMFM (+5.97%) ↑↑ WMA (+54.2%) ↑↑ WSBM (+770%) ↑↑ WTOT (+111%) The **control group** showed no significant changes.
**[21]** **Saudi Arabia**	39 children (age: 6–12; diagnosed with CP) **Swimming group—SG (*n* = 13):** Aquatic swimming intervention. **Aquatic Strengthening group—ASG (*n* = 13):** Aquatic Ai Chi stretching intervention. **Aquatic Play Control group (*n* =13):** Traditional land-based physiotherapy twice weekly. Assessments were conducted **before** treatment (baseline) and after an 8-week treatment period.	**Duration:** 8 weeks, 45 min/session **Frequency:** 3 sessions/w. **Exercises:** **SG:** 30 min program divided into: 10 min freestyle (coordination and cardiovascular fitness), 10 min breaststroke (upper/lower body strength), 10 min backstroke (postural alignment, shoulder/back strength). **ASG:** Three 10 min segments targeting: Upper limbs: dumbbells (0.5 kg), forearm rotations, elbow/shoulder exercises. Lower limbs: ankle cuff weights (0.5 kg), knee and hip strengthening. Trunk/neck: aqua floats/bars for flexion, extension, and rotation. **Aquatic Play Group:** 30 min play-based sessions (10 min each): Basketball, volleyball, and free water games for social and recreational engagement. **Setting:** Two Indoor heated pools. Muscle Power (MMT), Hand Grip Strength (HGS), Balance (MTUG), Endurance (1MWT), Functional Independence (FIM), Gross Motor Function (GMFCS) and Spasticity (MAS)	MMT: ↑↑↑ SG (+20%)–↑ ASG (+16%). HGS: ↑↑ SG (+14%)–↑ ASG (+25%). MTUG: ↑ SG (−26%) 1MWT: ↑ SG (+9%)–↑↑ ASG (+31%). FIM: ↑↑ SG & ASG in transportation and transfers. GMFCS: ↑ SG & ASG.
**[22]** **Poland**	22 adolescents (age: 14–16; diagnosed with DS) **Experimental group (*n* = 11):** water-based exercise and a swimming program. **Control group (*n* = 11):** Continued usual daily activities.	**Duration:** 33 weeks, 70–90 min/session **Frequency:** 2 sessions/w. **Exercises:** an aquatic protocol with two swimming coaches. **Setting:** Indoor heated pool (1.00–1.8 m depth). Muscle strength: Static arm strength, trunk strength and endurance/functional strength, Balance, flexibility, swimming skills: Water Orientation Test Alyn 2 (WOTA2) and Physical fitness was assessed using the Eurofit Test Battery (EfTB)	EfTB: ↑ Handgrip (+30.4%), ↑ Sit-ups 30 (21.6%) and ↑ Bent arm hang (106%). The balance, flexibility and plate tapping showed no significant changes. ↑↑↑ WMA (+302%) ↑↑↑ WSBM (+588%)
**[23]** **Iran & Chile**	30 young adults (age: 26.5–31.5 diagnosed with B) **Experimental group (*n* = 15):** aquatic exercise protocol. **Control group (*n* = 15):** No aquatic intervention; maintained usual activity. Assessments were pre-and post- intervention (8-week period).	**Duration:** 8 weeks, 60 min/session **Frequency:** 3 sessions/w. **Exercises:** Warm-up (5 min): increasing walking speed in water. Main aquatic exercises. Progression: Exercise difficulty increased over the 8 weeks by changing hand positions and increasing challenge (e.g., walking distance, tiptoe standing) to intensify proprioceptive/vestibular demand. **Setting:** Indoor heated pool. EMG activation (% of MVIC) in lower-limb muscles: Tibialis anterior (TA), Gastrocnemius medialis (GM), Rectus femoris (RF) and Biceps femoris (BF), Onset time (OT) of muscle activation (in ms) in the same muscles during AP and PA directions and Muscle strength (MS): measured (*n*/kg) for Ankle dorsiflexors, Ankle plantar-flexors, Knee flexors and Knee extensors.	EMG: ↑↑ TA (+18/25%)—↑↑GM (+15/20%) ↑ OT (−10/−20%) MS: ↑ Knee flexors (+10/15%)–↑ Knee extensors (+12/18%)
**[24]** **China**	42 children (age: 9–12; diagnosed with IDD) **Aquatic exercise group (*n* = 14).:** a combination of swimming skills and the Halliwick method. **Floor curling group (*n* = 14).** **Control group (*n* = 14**): free activities per week at a nearby sports field Assessments were conducted Baseline (pre-intervention) and after the 12-week intervention period.	**Duration:** 12 weeks, 60 min/session **Frequency:** 3 sessions/w. **Exercises:** Water-based balance/leg tasks or floor curling sliding/rolling tasks on land targeting legs. **Setting:** Indoor heated pool (26–27 °C, 1.00 m depth). Balance: Berg Balance Scale (BBS) and Lower limb muscle strength: handheld dynamometer (HHD). Hip extensor muscles, Hip flexor muscles, Knee extensor muscles, Knee flexor muscles, Toe extensor muscles and Ankle dorsiflexor muscles.	↑ BBS (+10.84%) ↑ HHD (+16.28%): ↑ Hip extensor and flexor muscles (+15/20%), ↑ Knee extensor and flexor muscles (+7/10%), ↑ Toe extensor muscles (15/20%) and ↑↑ Ankle dorsiflexor muscles (15/20%)

Abbreviations: 1MWT, 1 min walking test; 6MWT, 6-Minute Walk Test; ASG, Aquatic Strengthening group; ASD, Autism spectrum disorder; BS, Base of support; BBS, Berg Balance Scale; BG, Balance Group; BSCP, Bilateral spastic cerebral palsy; B, Blindness; CP, Cerebral Palsy; CHQ-PF50, Child Health Questionnaire-Parent Form 50; DS, Down Syndrome; DGI, Dynamic Gait Index; EMG, Electromyographic; EEI, Energy Expenditure Index; EfTB, Eurofit Test Battery; FIM, Functional Independence; GM, Gastrocnemius Medialis; GS, Gait speed; GMFM, Gross Motor Function Measure; HHD, Handheld Dynamometer; HGS, Hand Grip Strength; IDD, Intellectual disabilities; LF, Left Foot; MAS, Modified Ashworth Scale; MMT, Muscle Power; MS, Muscle Strength; OT, Onset Time; PGQ VAS, Parent-reported; PBS, Pediatric Balance Scale; RF, Right Foot; SG, Swimming Group; TA, Tibialis Anterior; TUG, Timed Up and Go; TCMS, Trunk Control Measurement Scale; TG, Trunk Group; VGAS, Visual Analog Scale for gait; WOTA2, Water Orientation Test Alyn 2; WMA, Water Mental adaptation; WSBM, Water Balance and Movement Skills; WTOT, Total aquatic skill score; WD, weight Distribution. * For time-based functional tests (e.g., TUG), lower scores indicate improved performance. Direction of change is reported accordingly throughout the table. ↑ improvement. For time-based outcomes, decreases indicate improvement. Statistical values are reported when available; otherwise, not reported (NR).

## Data Availability

No new data were created or analyzed in this study. Data sharing is not applicable to this article.

## Supplementary Materials

The following supporting information can be downloaded at https://www.mdpi.com/article/10.3390/healthcare14152256/s1: Supplementary File S1: Detailed Search Strategy for Each Database; Supplementary File S2: Reasons for Excluding Papers and References; Supplementary File S3: Risk of Bias Assessment for Each Study and Total Scores. Reference [41] is included in the Supplementary Materials.

## Abbreviations

The following abbreviations are used in this manuscript:

6MWT	Six-Minute Walk Test
ASD	Autism Spectrum Disorder
BBS	Berg Balance Scale
BSCP	Bilateral Spastic Cerebral Palsy
CP	Cerebral Palsy
DGI	Dynamic Gait Index
DS	Down Syndrome
DXA	Dual-energy X-ray Absorptiometry
EEI	Energy Expenditure Index
EMG	Electromyography
FIM	Functional Independence Measure
GMFCS	Gross Motor Function Classification System
GMFM	Gross Motor Function Measure
GRADE	Grading of Recommendations Assessment, Development and Evaluation
HGS	Hand Grip Strength
HM	Halliwick Method
IDD	Intellectual Disabilities
MVIC	Maximum Voluntary Isometric Contraction
MRI	Magnetic Resonance Imaging
MMT	Manual Muscle Test
PBS	Pediatric Balance Scale
PRISMA	Preferred Reporting Items for Systematic Reviews and Meta-Analyses
RCT	Randomized Controlled Trial
RoB2	Cochrane Risk of Bias 2 tool
TCMS	Trunk Control Measurement Scale
TUG	Timed Up and Go
WOTA 2	Water Orientation Test Alyn 2
