# Supplementary material for: Effects of Aquatic Exercise on Balance, Motor Function and Neuromuscular Performance in Children and Young Adults with Disabilities: A Systematic Review
Source: Healthcare (Basel). 2026 Jul 23;14(15):2256. doi: 10.3390/healthcare14152256 (PMC13465168; doi:10.3390/healthcare14152256)
Supplement: Supplementary file 1 [file healthcare-14-02256-s001.zip › Supplementary file S1.pdf]

Supplementary File S1: Detailed Search Strategy for Each Database

1. **PubMed**

("Halliwick" OR "aquatic therapy" OR "Halliwick aquatic therapy") AND ("muscle mass" OR "thickness" OR “strength”) AND ("children with disabilities" OR "children with special needs") AND ("physical exercise" OR "aquatic exercise")

2. MEDLINE

(TI("Halliwick" OR "aquatic therapy" OR "Halliwick aquatic therapy") OR AB("Halliwick" OR "aquatic therapy" OR "Halliwick aquatic therapy")) AND (TI ("muscle mass" OR "thickness") OR AB ("muscle mass" OR "thickness" OR “strength”)) AND (TI("children with disabilities" OR "children with special needs") OR AB("children with disabilities" OR "children with special needs")) AND (TI("physical exercise" OR "aquatic exercise") OR AB("physical exercise" OR "aquatic exercise"))

3. PEDro

Halliwick OR aquatic therapy OR hydrotherapy

aquatic therapy AND children

Method: clinical trial or randomised controlled trial or Systematic Review or Metha-analysis

4. Google Scholar

("Halliwick" OR "aquatic therapy" OR "Halliwick aquatic therapy") AND ("muscle mass" OR "thickness" OR “strength”) AND ("children with disabilities" OR "children with special needs") AND ("physical exercise" OR "aquatic exercise")

"Halliwick aquatic therapy" children disabilities muscle

"aquatic therapy" AND "muscle mass" AND children

5. Web Of Science

TS=("Halliwick" OR "aquatic therapy" OR "Halliwick aquatic therapy") AND TS=("muscle mass" OR "thickness") AND TS=("children with disabilities" OR "children with special needs") AND TS=("physical exercise" OR "aquatic exercise")
