# Supplementary material for: Effects of Aquatic Exercise on Balance, Motor Function and Neuromuscular Performance in Children and Young Adults with Disabilities: A Systematic Review
Source: Healthcare (Basel). 2026 Jul 23;14(15):2256. doi: 10.3390/healthcare14152256 (PMC13465168; doi:10.3390/healthcare14152256)
Supplement: Supplementary file 1 [file healthcare-14-02256-s001.zip › Supplementary file S2.pdf]

Supplementary File S2: Reasons for Excluding Papers and References

IDENTIFICATION OF STUDIES VIA DATABASES AND REGISTERS

REPORTS EXCLUDED (n= 191)

No intervention in children and young adult with disabilities (n = 15)(1–15)
No quantifiable data on muscle mass, strength or balance (n = 12)(16–27)
Non-aquatic interventions, no use of Halliwick or equivalent methodology (n = 7) (28–34)
Age not between 6 and 24 years old (n = 141) (35–176)
Samples with fewer than 10 participants (n = 6)(177–182)
Non English or spanish (n = 1)(183)
No RCT (n = 8)(184–191)
Non-original articles (letters, abstracts, comments) (n = 0)

1. Yu Z, Tang S, Hu M, Peng J, Fan Q, Leng L, et al. Aquatic High-Intensity Interval Training Improves Vascular Function, Whereas Aquatic Moderate-Intensity Continuous Training Lowers Resting Heart Rate in Overweight and Obese Young Adults: A Randomized Controlled Trial. J Sports Sci Med. 2025 Sept;24(3):666–75.

2. Liu W, Feng Z, Song S, Lei S. The effectiveness of physical activity in asthma management: An overview of systematic reviews. PLoS One. 2025;20(7):e0325488.

3. Chen S, Du L, Gao Y, Li H, Zheng Y, Xie L, et al. The applied study to improve the treatment of knee sports injuries in ultimate frisbee players based on personalized exercise prescription: a randomized controlled trial. Front Public Health. 2024;12:1441790.

4. Abdelrahman AY, El-Kosery SM, Abbassy AH, Botla AM. Effect of aquatic exercise versus aerobic exercise on primary dysmenorrhea and quality of life in adolescent females: A randomized controlled trial. Physiother Res Int. 2024 July;29(3):e2095.

5. Miguet M, Pereira B, Beaulieu K, Finlayson G, Mattosz P, Cardenoux C, et al. Effects of aquatic exercise on appetitive responses in adolescents with obesity: An exploratory study. Appetite. 2023 June 1;185:106540.

6. Elnaggar RK, Elfakharany MS. Aqua-Plyometric Exercises-Induced Changes in Muscle Strength, Bone Mineral Properties, and Physical Fitness in Patients With Juvenile Idiopathic Arthritis: A 12-Week, Randomized Controlled Trial. Pediatr Exerc Sci. 2023 Nov 1;35(4):198–205.

7. Peng MS, Wang R, Wang YZ, Chen CC, Wang J, Liu XC, et al. Efficacy of Therapeutic Aquatic Exercise vs Physical Therapy Modalities for Patients With

Chronic Low Back Pain: A Randomized Clinical Trial. JAMA Netw Open. 2022 Jan 4;5(1):e2142069.

8. Ansari S, AdibSaber F, Elmieh A, Gholamrezaei S. The effect of water-based intervention on sleep habits and two sleep-related cytokines in children with autism. Sleep Med. 2021 June;82:78–83.

9. Irandoust K, Taheri M, H'mida C, Neto GR, Trabelsi K, Ammar A, et al. Exergaming and Aquatic Exercises Affect Lung Function and Weight Loss in Obese Children. Int J Sports Med. 2021 June;42(6):566–72.

10. Kurobe K, Kousaka A, Ogita F, Matsumoto N. Metabolic responses to exercise on land and in water following glucose ingestion. Clin Physiol Funct Imaging. 2018 Mar;38(2):227–32.

11. Kim E, Kim T, Kang H, Lee J, Childers MK. Aquatic versus land-based exercises as early functional rehabilitation for elite athletes with acute lower extremity ligament injury: a pilot study. PM R. 2010 Aug;2(8):703–12.

12. Evcik D, Yigit I, Pusak H, Kavuncu V. Effectiveness of aquatic therapy in the treatment of fibromyalgia syndrome: a randomized controlled open study. Rheumatol Int. 2008 July;28(9):885–90.

13. Assis MR, Silva LE, Alves AMB, Pessanha AP, Valim V, Feldman D, et al. A randomized controlled trial of deep water running: Clinical effectiveness of aquatic exercise to treat fibromyalgia. Arthritis & Rheumatism. 2006 Feb 15;55(1):57–65.

14. Terrens AF, Soh SE, Morgan PE. The efficacy and feasibility of aquatic physiotherapy for people with Parkinson's disease: a systematic review. Disability and Rehabilitation. 2018 Nov 20;40(24):2847–56.

15. Gurpinar B, Kara B, Idiman E. Effects of aquatic exercises on postural control and hand function in Multiple Sclerosis: Halliwick versus Aquatic Plyometric Exercises: a randomised trial. J Musculoskelet Neuronal Interact. 2020;20(2):249–55.

16. Tapia C, Constanzo J, González V, Barría RM. The Effectiveness of Aquatic Therapy Based on the Halliwick Concept in Children with Cerebral Palsy: A Systematic Review. Developmental Neurorehabilitation. 2023 Oct 3;26(6–7):371–6.

17. Fragala-Pinkham M, Haley SM, O'Neil ME. Group aquatic aerobic exercise for children with disabilities. Dev Med Child Neurol. 2008 Nov;50(11):822–7.

18. Gorter JW, Currie SJ. Aquatic Exercise Programs for Children and Adolescents with Cerebral Palsy: What Do We Know and Where Do We Go? International Journal of Pediatrics. 2011;2011(1):712165.

19. Getz M, Hutzler Y, Vermeer A. Effects of aquatic interventions in children with neuromotor impairments: a systematic review of the literature. In: Database of Abstracts of Reviews of Effects (DARE): Quality-assessed Reviews [Internet] [Internet]. Centre for Reviews and Dissemination (UK); 2006 [cited 2025 Oct 21]. Available from: https://www.ncbi.nlm.nih.gov/books/NBK72491/

20. Akinola BI, Gbiri CA, Odebiyi DO. Effect of a 10-Week Aquatic Exercise Training Program on Gross Motor Function in Children With Spastic Cerebral Palsy. Glob Pediatr Health. 2019;6:2333794X19857378.

21. Bulut N, Yardimci BN, Ayvat E, Aran OT, Yilmaz Ö, Karaduman A. The effect of two different aerobic training modalities in a child with spinal muscular atrophy type II: a case report. J Exerc Rehabil. 2019 Apr;15(2):322–6.

22. (PDF) Assessing Aquatic Readiness as a Health-Enhancing Measure in Young Swimmers with Physical Disabilities: The Revised Aquatic Independence Measure (AIM-2). ResearchGate [Internet]. 2025 Oct 17 [cited 2025 Oct 24]; Available from: https://www.researchgate.net/publication/389844675_Assessing_Aquatic_Readiness_as_a_Health-Enhancing_Measure_in_Young_Swimmers_with_Physical_Disabilities_The_Revised_Aquatic_Independence_Measure_AIM-2

23. Chu CH, Pan CY. The effect of peer- and sibling-assisted aquatic program on interaction behaviors and aquatic skills of children with autism spectrum disorders and their peers/siblings. Research in Autism Spectrum Disorders. 2012 July 1;6(3):1211–23.

24. Redondo RV, Maldonado ELT, Maldonado JJV, Redondo RV, Maldonado ELT, Maldonado JJV. Efectividad de la terapia acuática en fisioterapia en pacientes pediátricos con problemas motores. Concepto halliwick. Revista Sanitaria de Investigación [Internet]. 2020 [cited 2025 Oct 21];1(2). Available from: https://portalcientifico.universidadeuropea.com/documentos/67f8011ecef0de3656894a72

25. Marzouki H, Soussi B, Selmi O, Hajji Y, Marsigliante S, Bouhlel E, et al. Effects of Aquatic Training in Children with Autism Spectrum Disorder. Biology (Basel). 2022 Apr 25;11(5):657.

26. Kemp E, Nikahd M, Howard M, Darragh A, Crasta JE. Improving water competency among children on the autism spectrum: the AquOTic randomized controlled trial. Front Pediatr [Internet]. 2024 Oct 7 [cited 2025 Oct 25];12. Available from: https://www.frontiersin.org/journals/pediatrics/articles/10.3389/fped.2024.1473328/full

27. Suarez-Villadat B, Luna-Oliva L, Acebes C, Villagra A. The effect of swimming program on body composition levels in adolescents with Down syndrome. Res Dev Disabil. 2020 July;102:103643.

28. Fiore P, Panza F, Cassatella G, Russo A, Frisardi V, Solfrizzi V, et al. Short-term effects of high-intensity laser therapy versus ultrasound therapy in the treatment of low back pain: a randomized controlled trial. Eur J Phys Rehabil Med. 2011 Sept;47(3):367–73.

29. Duchateau J, Baudry S. Insights into the neural control of eccentric contractions. Journal of Applied Physiology. 2014 June 1;116(11):1418–25.

30. Martín Rodríguez M. Efectos de un programa de fuerza en la participación de niños y niñas con trastornos del espectro autista. 2024;

31. Mahani MK, Karimloo M, Amirsalari S. Effects of Modified Adeli Suit Therapy on Improvement of Gross Motor Function in Children With Cerebral Palsy. Hong Kong Journal of Occupational Therapy [Internet]. 2011 June 1 [cited 2025 Oct 25];21. Available from: https://discovery.researcher.life/article/effects-of-modified-adeli-suit-therapy-on-improvement-of-gross-motor-function-in-children-with-cerebral-palsy/09ee7759d134331989433a8caacdfa88

32. Peñuela L, King G, Labbé A, Lui T, Maltais DB, Mérette C, et al. Children and Teens in Charge of Their Health (CATCH): A Feasibility Randomized Controlled Trial of Solution-Focused Coaching to Foster Healthy Lifestyles in Children with Spina Bifida or Cerebral Palsy. Developmental Neurorehabilitation [Internet]. 2025 Sept 18 [cited 2025 Oct 25];28. Available from: https://discovery.researcher.life/article/children-and-teens-in-charge-of-their-health-catch-a-feasibility-randomized-controlled-trial-of-solution-focused-coaching-to-foster-healthy-lifestyles-in-children-with-spina-bifida-or-cerebral-palsy/4d1461a272ba314cb99efb15e36b2aad

33. Huang HH, Hung CY, Kang LJ, Lai CL, Chang YC, Chen CL. Effects of standing ride-on car training on mobility and socialization in toddlers with mild and moderate motor delays: a pilot randomized controlled trial. Developmental Neurorehabilitation [Internet]. 2025 Aug 25 [cited 2025 Oct 25];28. Available from: https://discovery.researcher.life/article/effects-of-standing-ride-on-car-training-on-mobility-and-socialization-in-toddlers-with-mild-and-moderate-motor-delays-a-pilot-randomized-controlled-trial/98143b76d430379ab30850c43fe415fc

34. Herreros Laguía M. Efectividad del entrenamiento de fuerza en niños y jóvenes con parálisis cerebral. Revisión sistemática [Internet] [masterThesis]. Universidad Internacional de Andalucía; 2022 [cited 2025 Oct 21]. Available from: https://dspace.unia.es/handle/10334/7097

35. Kim S, Hsu FC, Groban L, Williamson J, Messier S. A pilot study of aquatic prehabilitation in adults with knee osteoarthritis undergoing total knee arthroplasty - short term outcome. BMC Musculoskelet Disord. 2021 Apr 26;22(1):388.

36. Kim Y, Vakula MN, Waller B, Bressel E. A systematic review and meta-analysis comparing the effect of aquatic and land exercise on dynamic balance in older adults. BMC Geriatr. 2020 Aug 25;20(1):302.

37. Shariat A, Ghayour Najafabadi M, Soroush Fard Z, Nakhostin-Ansari A, Shaw BS. A systematic review with meta-analysis on balance, fatigue, and motor function following aquatic therapy in patients with multiple sclerosis. Mult Scler Relat Disord. 2022 Dec;68:104107.

38. D'Cunha NM, Isbel S, McKune AJ, Kellett J, Naumovski N. Activities outside of the care setting for people with dementia: a systematic review. BMJ Open. 2020 Oct 6;10(10):e040753.

39. Tomschi F, Ransmann P, Hilberg T. Aerobic exercise in patients with haemophilia: A systematic review on safety, feasibility and health effects. Haemophilia. 2022 May;28(3):397–408.

40. Xavier DM, Abreu RAL, da Silva LF, Junior MG do N, Oliveira MX. Aquatic exercises for cystic fibrosis patients: A scoping review. J Bodyw Mov Ther. 2025 Oct;44:359–64.

41. Shi Z, Zhou H, Lu L, Pan B, Wei Z, Yao X, et al. Aquatic Exercises in the Treatment of Low Back Pain: A Systematic Review of the Literature and Meta-Analysis of Eight Studies. Am J Phys Med Rehabil. 2018 Feb;97(2):116–22.

42. Sadeghi Bahmani D, Motl RW, Razazian N, Khazaie H, Brand S. Aquatic exercising may improve sexual function in females with multiple sclerosis - an exploratory study. Mult Scler Relat Disord. 2020 Aug;43:102106.

43. Depiazzi J, Smith N, Gibson N, Wilson A, Langdon K, Hill K. Aquatic high intensity interval training to improve aerobic capacity is feasible in adolescents with cerebral palsy: pilot randomised controlled trial. Clin Rehabil. 2021 Feb;35(2):222–31.

44. Gu X, Zeng M, Cui Y, Fu J, Li Y, Yao Y, et al. Aquatic strength training improves postural stability and walking function in stroke patients. Physiother Theory Pract. 2023 Aug 3;39(8):1626–35.

45. Zhang Y, Wang YZ, Huang LP, Bai B, Zhou S, Yin MM, et al. Aquatic Therapy Improves Outcomes for Subacute Stroke Patients by Enhancing Muscular Strength of Paretic Lower Limbs Without Increasing Spasticity: A Randomized Controlled Trial. Am J Phys Med Rehabil. 2016 Nov;95(11):840–9.

46. Tomas-Carus P, Häkkinen A, Gusi N, Leal A, Häkkinen K, Ortega-Alonso A. Aquatic training and detraining on fitness and quality of life in fibromyalgia. Med Sci Sports Exerc. 2007 July;39(7):1044–50.

47. Yesil M, Ozcan O, Dundar U, Toktas H, Yesil H. Aquatic vs. land-based exercise after arthroscopic partial meniscectomy in middle-aged active patients

with a degenerative meniscal tear: A randomized, controlled study. J Orthop Sci. 2023 Mar;28(2):391–7.

48. Chen SC, Ding SB, Xie BC, Tian H, Lu CY. Are aquatic exercises efficacious in postmenopausal women with knee osteoarthritis? A meta-analysis of randomized controlled trials. J Sports Med Phys Fitness. 2019 Oct;59(10):1763–70.

49. Farrukh S, Habib S, Rafaqat A, Sarfraz A, Sarfraz Z, Tariq H. Association of exercise, brain-derived neurotrophic factor, and cognition among older women: A systematic review and meta-analysis. Arch Gerontol Geriatr. 2023 Nov;114:105068.

50. Faíl LB, Marinho DA, Marques EA, Costa MJ, Santos CC, Marques MC, et al. Benefits of aquatic exercise in adults with and without chronic disease-A systematic review with meta-analysis. Scand J Med Sci Sports. 2022 Mar;32(3):465–86.

51. Platz K, Kools S, Howie-Esquivel J. Benefits, Facilitators, and Barriers of Alternative Models of Cardiac Rehabilitation: A QUALITATIVE SYSTEMATIC REVIEW. J Cardiopulm Rehabil Prev. 2023 Mar 1;43(2):83–92.

52. Han J, Luan L, Adams R, Witchalls J, Newman P, Tirosh O, et al. Can Therapeutic Exercises Improve Proprioception in Chronic Ankle Instability? A Systematic Review and Network Meta-analysis. Arch Phys Med Rehabil. 2022 Nov;103(11):2232–44.

53. Andrade LS, Botton CE, David GB, Pinto SS, Häfele MS, Alberton CL. Cardiorespiratory Parameters Comparison Between Incremental Protocols Performed in Aquatic and Land Environments by Healthy Individuals: A Systematic Review and Meta-Analysis. Sports Med. 2022 Sept;52(9):2247–70.

54. Parfitt R, Hensman MY, Lucas SJE. Cerebral Blood Flow Responses to Aquatic Treadmill Exercise. Med Sci Sports Exerc. 2017 July;49(7):1305–12.

55. Dundar U, Solak O, Yigit I, Evcik D, Kavuncu V. Clinical effectiveness of aquatic exercise to treat chronic low back pain: a randomized controlled trial. Spine (Phila Pa 1976). 2009 June 15;34(14):1436–40.

56. Cadmus L, Patrick MB, Maciejewski ML, Topolski T, Belza B, Patrick DL. Community-based aquatic exercise and quality of life in persons with osteoarthritis. Med Sci Sports Exerc. 2010 Jan;42(1):8–15.

57. Qian Y, Fu X, Zhang H, Yang Y, Wang G. Comparative efficacy of 24 exercise types on postural instability in adults with Parkinson’s disease: a systematic review and network meta-analysis. BMC Geriatr. 2023 Aug 28;23(1):522.

58. Oh SJ, Lee SH. Comparing durability of water- and land-based exercise benefits among older adults in South Korea: A randomized controlled trial with 1-year follow-up. J Back Musculoskelet Rehabil. 2021;34(5):745–55.

59. Jiang L, Ding H, Ma Q, Gao S, Zhang X, Chun B. Comparing the effectiveness of different exercise interventions on quality of life in stroke patients: a randomized controlled network meta-analysis. BMC Neurol. 2025 Jan 17;25(1):24.

60. Chin ECY, Chung-Nam Lai S, Tsang SF, Ho-Ngai Chung S, Wong YL, Sran N, et al. Comparing the Effects of Aqua- and Land-Based Active Cooldown Exercises on Muscle Soreness and Sport Performance: A Randomized Crossover Study. Int J Sports Physiol Perform. 2024 Dec 1;19(12):1381–90.

61. Wang TJ, Lee SC, Liang SY, Tung HH, Wu SFV, Lin YP. Comparing the efficacy of aquatic exercises and land-based exercises for patients with knee osteoarthritis. J Clin Nurs. 2011 Sept;20(17–18):2609–22.

62. Gorman PH, Scott W, VanHiel L, Tansey KE, Sweatman WM, Geigle PR. Comparison of peak oxygen consumption response to aquatic and robotic therapy in individuals with chronic motor incomplete spinal cord injury: a randomized controlled trial. Spinal Cord. 2019 June;57(6):471–81.

63. Gusi N, Tomas-Carus P. Cost-utility of an 8-month aquatic training for women with fibromyalgia: a randomized controlled trial. Arthritis Res Ther. 2008;10(1):R24.

64. Zhang YH, Peng MS, Wang R, Wang YZ, Chen CC, Wang J, et al. Demographic and clinical characteristics associated with failure of physical therapy in chronic low back pain: a secondary analysis from a randomized controlled trial. Eur J Phys Rehabil Med. 2024 June;60(3):477–86.

65. Belza B, Topolski T, Kinne S, Patrick DL, Ramsey SD. Does adherence make a difference? Results from a community-based aquatic exercise program. Nurs Res. 2002;51(5):285–91.

66. Martínez-Carbonell Guillamón E, Burgess L, Immins T, Martínez-Almagro Andreo A, Wainwright TW. Does aquatic exercise improve commonly reported predisposing risk factors to falls within the elderly? A systematic review. BMC Geriatr. 2019 Feb 22;19(1):52.

67. Hall J, Swinkels A, Briddon J, McCabe CS. Does aquatic exercise relieve pain in adults with neurologic or musculoskeletal disease? A systematic review and meta-analysis of randomized controlled trials. Arch Phys Med Rehabil. 2008 May;89(5):873–83.

68. Deacon R, de Noronha M, Shanley L, Young K. Does the speed of aquatic therapy exercise alter arm volume in women with breast cancer related

lymphoedema? A cross-over randomized controlled trial. Braz J Phys Ther. 2019;23(2):140–7.

69. Patrick DL, Ramsey SD, Spencer AC, Kinne S, Belza B, Topolski TD. Economic evaluation of aquatic exercise for persons with osteoarthritis. Med Care. 2001 May;39(5):413–24.

70. Carrascosa MDC, Navas A, Artigues C, Ortas S, Portells E, Soler A, et al. Effect of aerobic water exercise during pregnancy on epidural use and pain: A multi-centre, randomised, controlled trial. Midwifery. 2021 Dec;103:103105.

71. Rewald S, Mesters I, Lenssen AF, Emans PJ, Wijnen W, de Bie RA. Effect of aqua-cycling on pain and physical functioning compared with usual care in patients with knee osteoarthritis: study protocol of a randomised controlled trial. BMC Musculoskelet Disord. 2016 Feb 18;17:88.

72. Dundar U, Solak O, Toktas H, Demirdal US, Subasi V, Kavuncu V, et al. Effect of aquatic exercise on ankylosing spondylitis: a randomized controlled trial. Rheumatol Int. 2014 Nov;34(11):1505–11.

73. Zhu H, Jin J, Zhao G. Effect of aquatic exercise on cardiovascular fitness in people with type 2 diabetes mellitus: a systematic review and meta-analysis. Kardiologiia. 2021 Sept 30;61(9):52–60.

74. Da Silva LA, Menguer L, Motta J, Dieke B, Mariano S, Tasca G, et al. Effect of aquatic exercise on mental health, functional autonomy, and oxidative dysfunction in hypertensive adults. Clin Exp Hypertens. 2018;40(6):547–53.

75. Oh S, Lee S. Effect of aquatic exercise on physical function and QOL in individuals with neurological disorder: A systematic review and meta-analysis. J Bodyw Mov Ther. 2021 July;27:67–76.

76. Braz de Oliveira MP, Rigo Lima C, da Silva SLA, Firmino Vaz Figueira EC, David Truax B, Smaili SM. Effect of aquatic exercise programs according to the International Classification of Functionality, Disability and Health domains in individuals with Parkinson’s disease: a systematic review and meta-analysis with GRADE quality assessment. Disabil Rehabil. 2024 Feb;46(3):429–42.

77. Kargarfard M, Etemadifar M, Baker P, Mehrabi M, Hayatbakhsh R. Effect of aquatic exercise training on fatigue and health-related quality of life in patients with multiple sclerosis. Arch Phys Med Rehabil. 2012 Oct;93(10):1701–8.

78. Saleh MSM, Rehab NI, Aly SMA. Effect of aquatic versus land motor dual task training on balance and gait of patients with chronic stroke: A randomized controlled trial. NeuroRehabilitation. 2019;44(4):485–92.

79. Huang L, Huang HL, Dang XW, Wang YJ. Effect of Body Weight Support Training on Lower Extremity Motor Function in Patients With Spinal Cord Injury: A

Systematic Review and Meta-analysis. Am J Phys Med Rehabil. 2024 Feb 1;103(2):149–57.

80. Oliveira GSD, Iraci L, Pinheiro GS, Casal MZ, Haas AN, Pochmann D, et al. Effect of exercise and grape juice on epigenetic modulation and functional outcomes in PD: A randomized clinical trial. Physiol Behav. 2020 Dec 1;227:113135.

81. Chen Y, Xu S, Shen J, Yang H, Xu W, Shao M, et al. Effect of Exercise on Fatigue in Multiple Sclerosis Patients: A Network Meta-analysis. Int J Sports Med. 2021 Dec;42(14):1250–9.

82. Waller B, Ogonowska-Slodownik A, Vitor M, Lambeck J, Daly D, Kujala UM, et al. Effect of therapeutic aquatic exercise on symptoms and function associated with lower limb osteoarthritis: systematic review with meta-analysis. Phys Ther. 2014 Oct;94(10):1383–95.

83. Carvalho RGS, Silva MF, Dias JM, Olkoski MM, Dela Bela LF, Pelegrinelli ARM, et al. Effectiveness of additional deep-water running for disability, lumbar pain intensity, and functional capacity in patients with chronic low back pain: A randomised controlled trial with 3-month follow-up. Musculoskelet Sci Pract. 2020 Oct;49:102195.

84. Avelar NCP, Bastone AC, Alcântara MA, Gomes WF. Effectiveness of aquatic and non-aquatic lower limb muscle endurance training in the static and dynamic balance of elderly people. Rev Bras Fisioter. 2010;14(3):229–36.

85. Kamioka H, Tsutani K, Okuizumi H, Mutoh Y, Ohta M, Handa S, et al. Effectiveness of aquatic exercise and balneotherapy: a summary of systematic reviews based on randomized controlled trials of water immersion therapies. J Epidemiol. 2010;20(1):2–12.

86. Barker AL, Talevski J, Morello RT, Brand CA, Rahmann AE, Urquhart DM. Effectiveness of aquatic exercise for musculoskeletal conditions: a meta-analysis. Arch Phys Med Rehabil. 2014 Sept;95(9):1776–86.

87. Lim JY, Tchai E, Jang SN. Effectiveness of aquatic exercise for obese patients with knee osteoarthritis: a randomized controlled trial. PM R. 2010 Aug;2(8):723–31; quiz 793.

88. Lu M, Su Y, Zhang Y, Zhang Z, Wang W, He Z, et al. Effectiveness of aquatic exercise for treatment of knee osteoarthritis: Systematic review and meta-analysis. Z Rheumatol. 2015 Aug;74(6):543–52.

89. Heywood S, McClelland J, Mentiplay B, Geigle P, Rahmann A, Clark R. Effectiveness of Aquatic Exercise in Improving Lower Limb Strength in Musculoskeletal Conditions: A Systematic Review and Meta-Analysis. Arch Phys Med Rehabil. 2017 Jan;98(1):173–86.

90. Duan X, Wei W, Zhou P, Liu X, Yu J, Xu Y, et al. Effectiveness of aquatic exercise in lower limb osteoarthritis: a meta-analysis of randomized controlled trials. Int J Rehabil Res. 2022 June 1;45(2):126–36.

91. Medrado LN, Mendonça MLM, Budib MB, Oliveira-Junior SA, Martinez PF. Effectiveness of aquatic exercise in the treatment of inflammatory arthritis: systematic review. Rheumatol Int. 2022 Oct;42(10):1681–91.

92. Taglietti M, Facci LM, Trelha CS, de Melo FC, da Silva DW, Sawczuk G, et al. Effectiveness of aquatic exercises compared to patient-education on health status in individuals with knee osteoarthritis: a randomized controlled trial. Clin Rehabil. 2018 June;32(6):766–76.

93. Kwok MMY, So BCL, Heywood S, Lai MCY, Ng SSM. Effectiveness of Deep Water Running on Improving Cardiorespiratory Fitness, Physical Function and Quality of Life: A Systematic Review. Int J Environ Res Public Health. 2022 Aug 1;19(15):9434.

94. Wheeler S, Acord-Vira A, Davis D. Effectiveness of Interventions to Improve Occupational Performance for People With Psychosocial, Behavioral, and Emotional Impairments After Brain Injury: A Systematic Review. Am J Occup Ther. 2016;70(3):7003180060p1-9.

95. de Souto Araujo ZT, de Miranda Silva Nogueira PA, Cabral EEA, de Paula Dos Santos L, da Silva IS, Ferreira GMH. Effectiveness of low-intensity aquatic exercise on COPD: a randomized clinical trial. Respir Med. 2012 Nov;106(11):1535–43.

96. Hernando-Garijo I, Jiménez-Del-Barrio S, Mingo-Gómez T, Medrano-de-la-Fuente R, Ceballos-Laita L. Effectiveness of non-pharmacological conservative therapies in adults with fibromyalgia: A systematic review of high-quality clinical trials. J Back Musculoskelet Rehabil. 2022;35(1):3–20.

97. Burton E, Farrier K, Hill KD, Codde J, Airey P, Hill AM. Effectiveness of peers in delivering programs or motivating older people to increase their participation in physical activity: Systematic review and meta-analysis. J Sports Sci. 2018 Mar;36(6):666–78.

98. Benzo-Iglesias MJ, Rocamora-Pérez P, Valverde-Martínez MÁ, García-Luengo AV, López-Liria R. Effectiveness of Water-Based Exercise in Patients with Chronic Obstructive Pulmonary Disease: Systematic Review and Meta-Analysis. Sensors (Basel). 2023 Oct 18;23(20):8557.

99. Delevatti RS, Kanitz AC, Bracht CG, Lisboa SDC, Marson EC, Reichert T, et al. Effects of 2 Models of Aquatic Exercise Training on Cardiorespiratory Responses of Patients With Type 2 Diabetes: The Diabetes and Aquatic Training Study-A Randomized Controlled Trial. J Phys Act Health. 2020 Sept 29;17(11):1091–9.

100. Casilda-López J, Valenza MC, Cabrera-Martos I, Díaz-Pelegrina A, Moreno-Ramírez MP, Valenza-Demet G. Effects of a dance-based aquatic exercise program in obese postmenopausal women with knee osteoarthritis: a randomized controlled trial. Menopause. 2017 July;24(7):768–73.

101. Waller B, Munukka M, Multanen J, Rantalainen T, Pöyhönen T, Nieminen MT, et al. Effects of a progressive aquatic resistance exercise program on the biochemical composition and morphology of cartilage in women with mild knee osteoarthritis: protocol for a randomised controlled trial. BMC Musculoskelet Disord. 2013 Mar 7;14:82.

102. Júnior RC, Terra AMSV, Gonçalves GCV, Costa MF, Pereira FC, Faria Dias MP, et al. Effects of an Aquatic Exercise Protocol on Blood Pressure, Respiratory Function, and Functional Capacity in Individuals with Type 2 Diabetes: A Randomized Controlled Trial. J Bodyw Mov Ther. 2025 Oct;44:97–105.

103. Aboarrage Junior AM, Teixeira CVLS, Dos Santos RN, Machado AF, Evangelista AL, Rica RL, et al. A High-Intensity Jump-Based Aquatic Exercise Program Improves Bone Mineral Density and Functional Fitness in Postmenopausal Women. Rejuvenation Res. 2018 Dec;21(6):535–40.

104. Ide MR, Belini MAV, Caromano FA. Effects of an aquatic versus non-aquatic respiratory exercise program on the respiratory muscle strength in healthy aged persons. Clinics (Sao Paulo). 2005 Apr;60(2):151–8.

105. Fernández García R, Sánchez Sánchez L de C, López Rodríguez MDM, Sánchez Granados G. [Effects of an exercise and relaxation aquatic program in patients with spondyloarthritis: A randomized trial]. Med Clin (Barc). 2015 Nov 6;145(9):380–4.

106. Kim JH, Jung YS, Kim JW, Ha MS, Ha SM, Kim DY. Effects of aquatic and land-based exercises on amyloid beta, heat shock protein 27, and pulse wave velocity in elderly women. Exp Gerontol. 2018 July 15;108:62–8.

107. Zhao P, Zhu G, Chen S, Pan Y, Chen K, Huang L, et al. Effects of Aquatic Exercise and Floor Curling on Balance Ability and Lower Limb Muscle Strength in Children with Intellectual Disabilities: A Pilot Study in China. Children. 2024 Jan;11(1):85.

108. Wang TJ, Belza B, Elaine Thompson F, Whitney JD, Bennett K. Effects of aquatic exercise on flexibility, strength and aerobic fitness in adults with osteoarthritis of the hip or knee. J Adv Nurs. 2007 Jan;57(2):141–52.

109. Silva LA da, Tortelli L, Motta J, Menguer L, Mariano S, Tasca G, et al. Effects of aquatic exercise on mental health, functional autonomy and oxidative stress in depressed elderly individuals: A randomized clinical trial. Clinics (Sao Paulo). 2019;74:e322.

110. Mattos F de, Leite N, Pitta A, Bento PCB. Effects of aquatic exercise on muscle strength and functional performance of individuals with osteoarthritis: a systematic review. Rev Bras Reumatol Engl Ed. 2016;56(6):530–42.

111. Prado AKG, Reichert T, Conceição MO, Delevatti RS, Kanitz AC, Kruel LFM. Effects of Aquatic Exercise on Muscle Strength in Young and Elderly Adults: A Systematic Review and Meta-Analysis of Randomized Trials. J Strength Cond Res. 2022 May 1;36(5):1468–83.

112. Li C, Khoo S, Adnan A. Effects of aquatic exercise on physical function and fitness among people with spinal cord injury: A systematic review. Medicine (Baltimore). 2017 Mar;96(11):e6328.

113. Buitrago-Restrepo CM, Patiño-Villada FA, Arango-Paternina CM. Effects of Aquatic Exercise on Physical Performance in Older Adults: A Systematic Review and Meta-Analysis. J Aging Phys Act. 2024 Oct 1;32(5):651–67.

114. Chen LJ, Fox KR, Ku PW, Chang YW. Effects of Aquatic Exercise on Sleep in Older Adults with Mild Sleep Impairment: a Randomized Controlled Trial. Int J Behav Med. 2016 Aug;23(4):501–6.

115. Katsura Y, Yoshikawa T, Ueda SY, Usui T, Sotobayashi D, Nakao H, et al. Effects of aquatic exercise training using water-resistance equipment in elderly. Eur J Appl Physiol. 2010 Mar;108(5):957–64.

116. Silva CT, Silva CK, Casonatto J, Carrasco AC, McVeigh JG, Cardoso JR. Effects of aquatic exercises in woman who have undergone surgery after breast cancer: a systematic review with meta-analysis. Braz J Phys Ther. 2025;29(5):101230.

117. Zhou WS, Mao SJ, Zhang SK, Xu H, Li WL. Effects of aquatic exercises on physical fitness and quality of life in postmenopausal women: an updated systematic review and meta-analysis. Front Public Health. 2023;11:1126126.

118. Ma J, Zhang T, Li X, Chen X, Zhao Q. Effects of aquatic physical therapy on clinical symptoms, physical function, and quality of life in patients with fibromyalgia: A systematic review and meta-analysis. Physiother Theory Pract. 2024 Feb;40(2):205–23.

119. Colado JC, Triplett NT, Tella V, Saucedo P, Abellán J. Effects of aquatic resistance training on health and fitness in postmenopausal women. Eur J Appl Physiol. 2009 May;106(1):113–22.

120. Pöyhönen T, Sipilä S, Keskinen KL, Hautala A, Savolainen J, Mälkiä E. Effects of aquatic resistance training on neuromuscular performance in healthy women. Med Sci Sports Exerc. 2002 Dec;34(12):2103–9.

121. Hande D, Shinde S, Dhumale A, Kale HY. Effects of Aquatic-Based Resistance, Balance, and Proprioceptive Training on Ankle-Foot Malalignments in Postmenopausal Obese Women. Cureus. 2025 July;17(7):e87571.

122. Silva MR, Alberton CL, Portella EG, Nunes GN, Martin DG, Pinto SS. Water-based aerobic and combined training in elderly women: Effects on functional capacity and quality of life. Exp Gerontol. 2018 June;106:54–60.

123. Costa RR, Pilla C, Buttelli ACK, Barreto MF, Vieiro PA, Alberton CL, et al. Water-Based Aerobic Training Successfully Improves Lipid Profile of Dyslipidemic Women: A Randomized Controlled Trial. Res Q Exerc Sport. 2018 June;89(2):173–82.

124. Häfele MS, Alberton CL, Häfele V, Schaun GZ, Nunes GN, Calonego C, et al. Water-Based Training Programs Improve Functional Capacity, Cognitive and Hemodynamic Outcomes? The ACTIVE Randomized Clinical Trial. Res Q Exerc Sport. 2023 Mar;94(1):24–34.

125. Monoli C, Tuhtan JA, Piccinini L, Galli M. Wearable technologies for monitoring aquatic exercises: A systematic review. Clin Rehabil. 2023 June;37(6):791–807.

126. Bergamin M, Ermolao A, Tolomio S, Berton L, Sergi G, Zaccaria M. Water-versus land-based exercise in elderly subjects: effects on physical performance and body composition. Clin Interv Aging. 2013;8:1109–17.

127. Waller B, Lambeck J, Daly D. Therapeutic aquatic exercise in the treatment of low back pain: a systematic review. Clin Rehabil. 2009 Jan;23(1):3–14.

128. Zão A, Cantista P. The role of land and aquatic exercise in ankylosing spondylitis: a systematic review. Rheumatol Int. 2017 Dec;37(12):1979–90.

129. Suntraluck S, Tanaka H, Suksom D. The Relative Efficacy of Land-Based and Water-Based Exercise Training on Macro- and Microvascular Functions in Older Patients With Type 2 Diabetes. J Aging Phys Act. 2017 July;25(3):446–52.

130. Yázigi F, Espanha M, Vieira F, Messier SP, Monteiro C, Veloso AP. The PICO project: aquatic exercise for knee osteoarthritis in overweight and obese individuals. BMC Musculoskelet Disord. 2013 Nov 13;14:320.

131. Rodríguez-Domínguez ÁJ, Rebollo-Salas M, Chillón-Martínez R, Rosales-Tristancho A, Villa-Del-Pino I, Jiménez-Rejano JJ. The most effective therapeutic exercises for pain intensity in women with fibromyalgia: A systematic review and network meta-analysis. Braz J Phys Ther. 2025;29(4):101226.

132. Zhang R, Li H, Kong T, Shan L, Wang P, Kang Y, et al. The impact of community-based, non-pharmaceutical interventions on anxiety and depression in fibromyalgia: A systematic review and network meta-analysis. J Psychiatr Res. 2025 Feb;182:50–8.

133. Farinha C, Santos H, Serrano J, Oliveiros B, Silva FM, Cascante-Rusenhack M, et al. The Impact of Aquatic Exercise Programs on the Intima-Media thickness of the Carotid Arteries, Hemodynamic Parameters, Lipid Profile and Chemokines of Community-Dwelling Older Persons: A Randomized Controlled Trial. Int J Environ Res Public Health. 2022 Mar 13;19(6):3377.

134. Araújo JP, Neto GR, Loenneke JP, Bemben MG, Laurentino GC, Batista G, et al. The effects of water-based exercise in combination with blood flow restriction on strength and functional capacity in post-menopausal women. Age (Dordr). 2015 Dec;37(6):110.

135. Hong K, Esquivel JH. The Effects of Nonconventional Exercise on Functional Capacity and Quality of Life in Patients With Heart Failure: A Systematic Review. J Cardiovasc Nurs. 2022 Dec 1;37(6):530–45.

136. Nishiwaki M, Kawakami R, Saito K, Tamaki H, Ogita F. The effects of exercise training under mild hypoxic conditions on body composition and circulating adiponectin in postmenopausal women. Clin Physiol Funct Imaging. 2016 Nov;36(6):468–75.

137. Gunay SM, Keser I, Bicer ZT. The effects of balance and postural stability exercises on spa based rehabilitation programme in patients with ankylosing spondylitis. J Back Musculoskelet Rehabil. 2018;31(2):337–46.

138. Alikhajeh Y, Afroundeh R, Mohammad Rahimi GR, Bayani B. The Effects of Aquatic Exercise Training on Functional and Hemodynamic Responses in Patients With Heart Failure: A Systematic Review and Meta-Analysis. Biol Res Nurs. 2025 Jan;27(1):127–41.

139. Júnior FA, Gomes SG, da Silva FF, Souza PM, Oliveira EC, Coelho DB, et al. The effects of aquatic and land exercise on resting blood pressure and post-exercise hypotension response in elderly hypertensives. Cardiovasc J Afr. 2020 June 23;31(3):116–22.

140. Cantarero-Villanueva I, Fernández-Lao C, Cuesta-Vargas AI, Del Moral-Avila R, Fernández-de-Las-Peñas C, Arroyo-Morales M. The effectiveness of a deep water aquatic exercise program in cancer-related fatigue in breast cancer survivors: a randomized controlled trial. Arch Phys Med Rehabil. 2013 Feb;94(2):221–30.

141. Igarashi Y, Nogami Y. The effect of regular aquatic exercise on blood pressure: A meta-analysis of randomized controlled trials. Eur J Prev Cardiol. 2018 Jan;25(2):190–9.

142. Murtezani A, Nevzati A, Ibraimi Z, Sllamniku S, Meka VS, Abazi N. The effect of land versus aquatic exercise program on bone mineral density and physical function in postmenopausal women with osteoporosis: a randomized controlled trial. Ortop Traumatol Rehabil. 2014;16(3):319–25.

143. Amedoro A, Berardi A, Conte A, Pelosin E, Valente D, Maggi G, et al. The effect of aquatic physical therapy on patients with multiple sclerosis: A systematic review and meta-analysis. Mult Scler Relat Disord. 2020 June;41:102022.

144. Giuriati S, Servadio A, Temperoni G, Curcio A, Valente D, Galeoto G. The effect of aquatic physical therapy in patients with stroke: A systematic review and meta-analysis. Top Stroke Rehabil. 2021 Jan;28(1):19–32.

145. Irandoust K, Taheri M, Mirmoezzi M, H'mida C, Chtourou H, Trabelsi K, et al. The Effect of Aquatic Exercise on Postural Mobility of Healthy Older Adults with Endomorphic Somatotype. Int J Environ Res Public Health. 2019 Nov 10;16(22):4387.

146. Waller B, Ogonowska-Słodownik A, Vitor M, Rodionova K, Lambeck J, Heinonen A, et al. The effect of aquatic exercise on physical functioning in the older adult: a systematic review with meta-analysis. Age Ageing. 2016 Sept;45(5):593–601.

147. Arnold CM, Faulkner RA. The effect of aquatic exercise and education on lowering fall risk in older adults with hip osteoarthritis. J Aging Phys Act. 2010 July;18(3):245–60.

148. Moreira LDF, Fronza FCAO, Dos Santos RN, Zach PL, Kunii IS, Hayashi LF, et al. The benefits of a high-intensity aquatic exercise program (HydrOS) for bone metabolism and bone mass of postmenopausal women. J Bone Miner Metab. 2014 July;32(4):411–9.

149. Muñoz-Gómez E, Arnal-Gómez A, López Cascón A, Espí-López GV. Systematic review of aquatic therapeutic exercise efficacy in breast cancer survivors. Support Care Cancer. 2022 Dec 16;31(1):44.

150. Batterham SI, Heywood S, Keating JL. Systematic review and meta-analysis comparing land and aquatic exercise for people with hip or knee arthritis on function, mobility and other health outcomes. BMC Musculoskelet Disord. 2011 June 2;12:123.

151. Heywood S, McClelland J, Geigle P, Rahmann A, Clark R. Spatiotemporal, kinematic, force and muscle activation outcomes during gait and functional exercise in water compared to on land: A systematic review. Gait Posture. 2016 July;48:120–30.

152. Chien KY, Chen WC, Kan NW, Hsu MC, Lee SL. Responses of blood pressure and lactate levels to various aquatic exercise movements in postmenopausal women. J Sports Med Phys Fitness. 2015 Dec;55(12):1423–30.

153. Igarashi Y, Nogami Y. Response of Lipids and Lipoproteins to Regular Aquatic Endurance Exercise: A Meta-Analysis of Randomized Controlled Trials. J Atheroscler Thromb. 2019 Jan 1;26(1):14–30.

154. Hoshi D, Fukuie M, Tomoto T, Qin W, Tarumi T, Sugawara J, et al. Respiratory regulation and lung volume during aquatic and land-based exercise in healthy Young adults. Physiol Rep. 2025 Oct;13(19):e70564.

155. Pechter Ü, Raag M, Ots-Rosenberg M. Regular aquatic exercise for chronic kidney disease patients: a 10-year follow-up study. Int J Rehabil Res. 2014 Sept;37(3):251–5.

156. Kargarfard M, Shariat A, Ingle L, Cleland JA, Kargarfard M. Randomized Controlled Trial to Examine the Impact of Aquatic Exercise Training on Functional Capacity, Balance, and Perceptions of Fatigue in Female Patients With Multiple Sclerosis. Arch Phys Med Rehabil. 2018 Feb;99(2):234–41.

157. Rodríguez-Blanque R, Sanchez-Garcia JC, Sanchez-Lopez AM, Expósito-Ruiz M, Aguilar-Cordero MJ. Randomized Clinical Trial of an Aquatic Physical Exercise Program During Pregnancy. J Obstet Gynecol Neonatal Nurs. 2019 May;48(3):321–31.

158. Häfele MS, Alberton CL, Schaun GZ, Häfele V, Nunes GN, Andrade LS, et al. Quality of life responses after combined and aerobic water-based training programs in older women: a randomized clinical trial (ACTIVE Study). Aging Clin Exp Res. 2022 May;34(5):1123–31.

159. Marçal IR, Fernandes B, do Amaral VT, Pelaquim RL, Ciolac EG. Prescribing and Self-Regulating Heated Water-Based Exercise by Rating of Perceived Exertion in Older Individuals With Hypertension. J Aging Phys Act. 2022 Oct 1;30(5):747–52.

160. Suomi R, Koceja DM. Postural sway characteristics in women with lower extremity arthritis before and after an aquatic exercise intervention. Arch Phys Med Rehabil. 2000 June;81(6):780–5.

161. Cunha RM, Costa AM, Silva CNF, Póvoa TIR, Pescatello LS, Lehnen AM. Postexercise Hypotension After Aquatic Exercise in Older Women With Hypertension: A Randomized Crossover Clinical Trial. Am J Hypertens. 2018 Jan 12;31(2):247–52.

162. Muthukrishan R, Badr Ul Islam FM, Shanmugam S, Arulsingh W, Gopal K, Kandakurti PK, et al. Perturbation-based Balance Training in Adults Aged Above 55 Years with Chronic Low Back Pain: A Comparison of Effects of Water versus Land Medium - A Preliminary Randomized Trial. Curr Aging Sci. 2024;17(2):156–68.

163. Sousa AP de, Almeida LA, Lourenço BP, Alvares LD, Avila MA. Pain neuroscience education improves quality of life when added to aquatic exercise therapy for women with fibromyalgia: randomized controlled clinical trial. Disabil Rehabil. 2024 Apr;46(8):1559–69.

164. Cheng M, Ni J, Liu F, Wang S. Optimal type and dose of exercise to improve fall behavior in older adults: a systematic evaluation and network meta-analysis. Ageing Res Rev. 2025 Oct 17;102924.

165. Fan X, Yuan Y, Bai Y, Cheng C, Wang J, Wang T, et al. Optimal dose and type of exercise improve the overall balance in adults with Parkinson's disease: a systematic review and Bayesian network meta-analysis. Neurol Sci. 2025 Sept;46(9):4169–80.

166. Ferreira RM, Duarte JA, Gonçalves RS. Non-pharmacological and non-surgical interventions to manage patients with knee osteoarthritis: An umbrella review. Acta Reumatol Port. 2018;43(3):182–200.

167. Adsett JA, Morris NR, Kuys SS, Paratz JD, Mudge AM. Motivators and barriers for participation in aquatic and land-based exercise training programs for people with stable heart failure: A mixed methods approach. Heart Lung. 2019;48(4):287–93.

168. Reichert T, Delevatti RS, Prado AKG, Bagatini NC, Simmer NM, Meinerz AP, et al. Low- and High-Volume Water-Based Resistance Training Induces Similar Strength and Functional Capacity Improvements in Older Women: A Randomized Study. J Phys Act Health. 2018 Aug 1;15(8):592–9.

169. Park HK, Lee HJ, Lee SJ, Lee WH. Land-based and aquatic trunk exercise program improve trunk control, balance and activities of daily living ability in stroke: a randomized clinical trial. Eur J Phys Rehabil Med. 2019 Dec;55(6):687–94.

170. Naylor LH, Maslen BA, Cox KL, Spence AL, Robey E, Haynes A, et al. Land-versus water-walking interventions in older adults: Effects on body composition. J Sci Med Sport. 2020 Feb;23(2):164–70.

171. Dong R, Wu Y, Xu S, Zhang L, Ying J, Jin H, et al. Is aquatic exercise more effective than land-based exercise for knee osteoarthritis? Medicine (Baltimore). 2018 Dec;97(52):e13823.

172. Aidar FJ, Gama de Matos D, de Souza RF, Gomes AB, Saavedra F, Garrido N, et al. Influence of aquatic exercises in physical condition in patients with multiple sclerosis. J Sports Med Phys Fitness. 2018 May;58(5):684–9.

173. Ay A, Yurtkuran M. Influence of aquatic and weight-bearing exercises on quantitative ultrasound variables in postmenopausal women. Am J Phys Med Rehabil. 2005 Jan;84(1):52–61.

174. Lv X, Wang J, Bao Y, Tang Y, Xing W, Wu Q, et al. The effectiveness of balneotherapy and aquatic exercise on bone metabolism: A systematic review and meta-analysis. Complement Ther Clin Pract. 2021 Aug;44:101429.

175. Delevatti RS, Santiago É, Kanitz AC, Marson EC, Reichert T, de M Bones V, et al. Improving the neuromuscular fitness of patients with type 2 diabetes by aquatic exercise training - The Diabetes and Aquatic Training Study (DATS 3): a randomized controlled trial. J Sports Med Phys Fitness. 2024 Oct;64(10):1098–106.

176. Dunlap E, Alhalimi T, McLaurin N, Tanaka H. Impact of Land Versus Water Environments on Orthostatic Hypotension in Older Adults: A Randomized Crossover Study. J Aging Phys Act. 2025 Feb 1;33(1):35–41.

177. Vodakova E, Chatziioannou D, Jesina O, Kudlacek M. The Effect of Halliwick Method on Aquatic Skills of Children with Autism Spectrum Disorder. IJERPH. 2022 Dec 5;19(23):16250.

178. Thorpe DE, Reilly M, Case L. The Effects of an Aquatic Resistive Exercise Program on Ambulatory Children with Cerebral Palsy. The Journal of Aquatic Physical Therapy. 2005;13(2):21.

179. Ballaz L, Plamondon S, Lemay M. Group aquatic training improves gait efficiency in adolescents with cerebral palsy. Disability and Rehabilitation. 2011 Jan;33(17–18):1616–24.

180. Ballington SJ, Naidoo R. The carry-over effect of an aquatic-based intervention in children with cerebral palsy. Afr J Disabil. 2018 Oct 29;7(0):361.

181. Koumenidou M, Kotzamanidou MC, Panoutsakopoulos V, Siaperas P, Misailidou V, Tsalis GA. The Long-Term Adaptations of a Combined Swimming and Aquatic Therapy Intervention in an Adult Person with High-Functioning Autism (Asperger's Syndrome): A Case Study. Healthcare. 2023 Jan;11(22):2986.

182. Esmailiyan M, Marandi SM, Darvishi M, Haghjooy Javanmard S, Amerizadeh A. The Effect of Eight Weeks of Aquatic Exercises on Muscle Strength in Children with Cerebral Palsy: A Case Study. Adv Biomed Res. 2023 Apr 25;12:87.

183. 廖婷丛芳, LIAO Ting CF. 残疾儿童水中运动康复的健康与功能效益：基于ICD-11和ICF的系统综述的系统综述. 《中国康复理论与实践》. 2023 Nov 30;29(11):1286–95.

184. Ogonowska-Slodownik A, Jakobowicz O, Alexander L, Marinho-Buzelli AR, Devion C, Morgulec-Adamowicz N. Aquatic Therapy in Children and Adolescents with Disabilities: A Scoping Review. Children (Basel). 2024 Nov 20;11(11):1404.

185. Xiang A, Fu Y, Wang C, Huang D, Qi J, Zhao R, et al. Aquatic therapy for spastic cerebral palsy: a scoping review. Eur J Med Res. 2024 Nov 29;29(1):569.

186. Cole AJ, Becker BE. Comprehensive Aquatic Therapy. Butterworth-Heinemann; 2004. 392 p.

187. Shariat A, Najafabadi MG, Dos Santos IK, Anastasio AT, Milajerdi HR, Hassanzadeh G, et al. The Effectiveness of Aquatic Therapy on Motor and Social Skill as Well as Executive Function in Children With Neurodevelopmental Disorder: A Systematic Review and Meta-analysis. Arch Phys Med Rehabil. 2024 May;105(5):1000–7.

188. Tao Y, Cao Z, Shin MC, Chen M, Han S. The effects of hydrotherapy on athletic ability in children with cerebral palsy: A systematic review and meta-analysis. PLOS ONE. 2025 June 10;20(6):e0325517.

189. Kelly M, Darrah J. Aquatic exercise for children with cerebral palsy. Dev Med Child Neurol. 2005 Dec;47(12):838–42.

190. Li F, Huang W, Li H, Liu Z, Feng L. Rehabilitation effect of aquatic exercise on motor function in children with cerebral palsy: Systematic review of randomized controlled trials. J Back Musculoskelet Rehabil. 2025 July;38(4):662–73.

191. Jorgic B. The Effects of a Program of Swimming and Aquatic Exercise on Flexibility in Children with Cerebral Palsy. Facta Universitatis Series Physical Education and Sport [Internet]. 2014 Oct 16 [cited 2025 Oct 24]; Available from: https://www.academia.edu/76773364/The_Effects_of_a_Program_of_Swimming_and_Aquatic_Exercise_on_Flexibility_in_Children_with_Cerebral_Palsy
