# Supplementary material for: Effects of Aquatic Exercise on Balance, Motor Function and Neuromuscular Performance in Children and Young Adults with Disabilities: A Systematic Review
Source: Healthcare (Basel). 2026 Jul 23;14(15):2256. doi: 10.3390/healthcare14152256 (PMC13465168; doi:10.3390/healthcare14152256)
Supplement: Supplementary file 1 [file healthcare-14-02256-s001.zip › Supplementary file S3.pdf]

Supplementary File S3: Risk of Bias Assessment for Each Study and Total Scores

Risk of Bias 2
Table assessment

Reference: ANSARI ET AL - 2021

Variable assessed: xxxx
Page: xxxx **Figure/Table:** xxxx
Assessor name: Francisco Javier Cánovas Hernández
Date: 31/10/2025

Summary Table

Study reference	Study design	Domain 1	Domain 2	Domain 3	Domain 4	Domain 5	Overall
ANSARI ET AL - 2021	RCT	●○○ Low risk of bias	●○○ Low risk of bias	●○○ Low risk of bias	●○○ Low risk of bias	●○○ Low risk of bias	●○○ Low risk of bias

●●●High risk of bias, ●●○ Some concerns, ●○○Low risk of bias

Domain 1: Random sequence generation; **Domain 2:** Deviations from the intended intervention; **Domain 3:** Missing outcome data; **Domain 4:** Measurement of the outcome; **Domain 5:** Selection of reported result

Assessment Table

N tot	N int	N con	Time int	Participants	Interventions	Comparator	Outcome	Finding
xxxx	xxxx	xxxx	xxxx	xxxx	xxxx	xxxx	xxxx	xxxx

Outcome variable	Device	Partial Reliability	Absolute Reliability	Other procedures characteristics
xxxx	xxxx	xxxx	xxxx	xxxx

Study characteristics

Bias Domain	Judgement	Q		Support for judgement
**Random sequence generation**	●○○ Low risk of bias	1.1	Y/PY	“were randomly divided into three groups”
		1.2	Y/PY	“using a randomized block design”
		1.3	PN/N	“including the kata techniques training group (N = 10), aquatic exercise training group (N = 10), and control group (N = 10)”
**Deviations from the intended intervention**	●○○ Low risk of bias	2.1	Y/PY/NI	“All participants were screened by an experienced physician whether have other medical conditions that limited their physical activity capacities”
		2.2	Y/PY/NI	“All participants were screened by an experienced physician whether have other medical conditions that limited their physical activity capacities”
		2.3	PN/N	“…to be eligible for performing the exercise training protocols.”
		2.4	NA/Y/PY/PN/N/NI	-
		2.5	NA/Y/PY/PN/N/NI	-
		2.6	Y/PY	“There were two assessments before the intervention (T1: baseline) and after the intervention (T2: post-intervention), in which participants’ static and dynamic balance were evaluated.”
		2.7	NA/Y/PY/PN/N/NI	-
**Missing outcome data**	●○○ Low risk of bias	3.1	Y/PY/PN/N/NI	“…information for the three groups at baseline measurement.”
		3.2	NA/Y/PY/PN/N	-
		3.3	NA/Y/PY/PN/N/NI	-
		3.4	NA/Y/PY/PN/N/NI	-
**Measurement of the outcome**	●○○ Low risk of bias	4.1	PN/N	“There was no statistically significant difference in any demographic variables including age, height, weight, BMI, and autism severity at the beginning of the intervention.”
		4.2	PN/N	“Static and dynamic balance values in three research groups”
		4.3	Y/PY/NI	It does not state this explicitly, but it is implied.
		4.4	PN/N	“…were used to check the normality and homogeneity of variances of the data, respectively; and ANCOVA was utilized to evaluate the research hypotheses, as the data were normally distributed.”
		4.5	NA/Y/PY/PN/N/NI	-
**Selection of the reported results**	●○○ Low risk of bias	5.1	PN/N	“The modified stork standing test was used to measure static balance.” “To assess dynamic balance, the heel-to-toe walking test was used.”
		5.2	PN/N	“Table 3 Static and dynamic balance values in three research groups”

		5.3	PN/N	"The modified stork standing test was used to measure static balance." "To assess dynamic balance, the heel-to-toe walking test was used."
**OVERALL***	●○○<br>Low risk of bias		●●●<br>High risk of bias	The study is judged to be at high risk of bias in at least one domain for this result OR the study is judged to have some concerns for multiple domains in a way that substantially lowers confidence in the result.
			●●○<br>Some concerns	The study is judged to raise some concerns in at least one domain for this result, but not to be at high risk of bias for any domain
			●○○<br>Low risk of bias	The study is judged to be at low risk of bias for all domains for this result.

*Reference: Sterne, J. A., Savović, J., Page, M. J., Elbers, R. G., Blencowe, N. S., Boutron, I., & Higgins, J. P. (2019). RoB 2: a revised tool for assessing risk of bias in randomised trials. *bmj*, *366*.

Reference: CASTRO ET AL - 2025

Variable assessed: xxxx
Page: xxxx **Figure/Table:** xxxx
Assessor name: Francisco Javier Cánovas Hernández
Date: 31/10/2025

Summary Table

Study reference	Study design	Domain 1	Domain 2	Domain 3	Domain 4	Domain 5	Overall
CASTRO ET AL - 2025	RCT	●○○ Low risk of bias	●○○ Low risk of bias	●○○ Low risk of bias	●○○ Low risk of bias	●○○ Low risk of bias	●○○ Low risk of bias

●●●High risk of bias, ●●○ Some concerns, ●○○Low risk of bias

Domain 1: Random sequence generation; **Domain 2:** Deviations from the intended intervention; **Domain 3:** Missing outcome data; **Domain 4:** Measurement of the outcome; **Domain 5:** Selection of reported result

Assessment Table

N tot	N int	N con	Time int	Participants	Interventions	Comparator	Outcome	Finding
xxxx	xxxx	xxxx	xxxx	xxxx	xxxx	xxxx	xxxx	xxxx

Outcome variable	Device	Partial Reliability	Absolute Reliability	Other procedures characteristics
xxxx	xxxx	xxxx	xxxx	xxxx

Study characteristics

Bias Domain	Judgement	Q		Support for judgement
**Random sequence generation**	●○○ Low risk of bias	1.1	Y/PY	"The assessments were carried out by an independent and blinded researcher"
		1.2	Y/PY	"Another researcher was responsible for conducting randomization, concealment, and allocation."
		1.3	PN/N	"…and selected 16 children (8 allocated to each group)"
**Deviations from the intended intervention**	●○○ Low risk of bias	2.1	PN/N	"This is a 2 arm, parallel, randomized, controlled, and blinded clinical trial." "All assessments were performed by a single experienced pediatric physical therapist who was not involved in the intervention procedures and had extensive training in the administration of all outcome measures."
		2.2	Y/PY	"Another 5 researchers with more than 5 years of experience in treating children with CP in aquatic therapy were responsible for carrying out the interventions."
		2.3	PN/N	"The participants' level of collaboration and understanding of simple movement commands was observed"
		2.4	NA/Y/PY/PN/N/NI	-
		2.5	NA/Y/PY/PN/N/NI	-
		2.6	Y/PY	"The measurement tools used in this study have established validity and high intra- and inter-rater reliability in pediatric populations, as cited in the respective validation studies."
		2.7	NA/Y/PY/PN/N/NI	-
**Missing outcome data**	●○○ Low risk of bias	3.1	Y/PY/PN/N/NI	"The assessments followed the same sequence for all participants" "all assessments followed a standardized protocol and were conducted in the same order and conditions for all participants." "totaling a sample of 16 individuals, randomly allocated to 2 intervention groups"
		3.2	NA/Y/PY/PN/N	-
		3.3	NA/Y/PY/PN/N/NI	-
		3.4	NA/Y/PY/PN/N/NI	-
**Measurement of the outcome**	●○○ Low risk of bias	4.1	PN/N	"The measurement tools used in this study have established validity and high intra- and inter-rater reliability in pediatric populations, as cited in the respective validation studies."
		4.2	PN/N	"all assessments followed a standardized protocol and were conducted in the same order and conditions for all participants."
		4.3	PN/N	"All assessments were performed by a single experienced pediatric physical therapist who was not involved in the intervention procedures and had extensive training in the administration of all outcome measures."

		4.4	NA/Y/PY/PN/N/NI	-
		4.5	NA/Y/PY/PN/N/NI	-
**Selection of the reported results**	●○○<br>Low risk of bias	5.1	Y/PY	"In the intragroup comparison analysis of outcomes with symmetrical distribution, both groups had a significant difference<br>between the means after the intervention, for all comparisons"
		5.2	PN/N	"Static sitting balance, dynamic<br>sitting balance, and balance reactions." "Pediatric Balance Scale<br>(PBS)" They measured the balance and that was what they used.
		5.3	PN/N	"A total of 300 individuals were screened, 68 were included based on diagnostic of bilateral spastic CP classified to GMFCS<br>II or III and of these, 52 were excluded, totaling a sample of 16<br>individuals, randomly allocated to 2 intervention groups"<br>Required number of measurements per group
**OVERALL***	●○○<br>Low risk of bias		●●●<br>High risk of bias	The study is judged to be at high risk of bias in at least one domain for this result OR the study is judged to have some concerns for multiple domains in a way that substantially lowers confidence in the result.
			●●○<br>Some concerns	The study is judged to raise some concerns in at least one domain for this result, but not to be at high risk of bias for any domain
			●○○<br>Low risk of bias	The study is judged to be at low risk of bias for all domains for this result.

*Reference: Sterne, J. A., Savović, J., Page, M. J., Elbers, R. G., Blencowe, N. S., Boutron, I., & Higgins, J. P. (2019). RoB 2: a revised tool for assessing risk of bias in randomised trials. *bmj*, *366*.

Reference: CHANDOLIAS ET AL - 2022

Variable assessed: xxxx
Page: xxxx **Figure/Table:** xxxx
Assessor name: Francisco Javier Cánovas Hernández
Date: 31/10/2025

Summary Table

Study reference	Study design	Domain 1	Domain 2	Domain 3	Domain 4	Domain 5	Overall
CHANDOLIAS ET AL - 2022	RCT	●●● High risk of bias	●○○ Low risk of bias	●○○ Low risk of bias	●○○ Low risk of bias	●○○ Low risk of bias	●●○ Some concerns

●●●High risk of bias, ●●○ Some concerns, ●○○Low risk of bias

Domain 1: Random sequence generation; **Domain 2:** Deviations from the intended intervention; **Domain 3:** Missing outcome data; **Domain 4:** Measurement of the outcome; **Domain 5:** Selection of reported result

Assessment Table

N tot	N int	N con	Time int	Participants	Interventions	Comparator	Outcome	Finding
xxxx	xxxx	xxxx	xxxx	xxxx	xxxx	xxxx	xxxx	xxxx

Outcome variable	Device	Partial Reliability	Absolute Reliability	Other procedures characteristics
xxxx	xxxx	xxxx	xxxx	xxxx

Study characteristics

Bias Domain	Judgement	Q		Support for judgement
**Random sequence generation**	●●● High risk of bias	1.1	Y/PY/PN/N/NI	-
		1.2	NI	No information
		1.3	Y/PY	"The 10 children attended a Hydrotherapy program according to the Halliwick philosophy by specially trained method therapists. The 6 children attended treatment with the methods of classical physiotherapy"
**Deviations from the intended intervention**	●○○ Low risk of bias	2.1	Y/PY	"The children who participated in the study knew their therapists from the beginning of the program."
		2.2	Y/PY	"The children who participated in the study knew their therapists from the beginning of the program."
		2.3	PN/N	"There are no incomplete observations in the sample, so these two columns are identical."
		2.4	NA/Y/PY/PN/N/NI	-
		2.5	NA/Y/PY/PN/N/NI	-
		2.6	Y/PY	"For the needs of the research, the balance of the children was evaluated with the assessment tools Berg, GMFM, and footprint plate."
		2.7	NA/Y/PY/PN/N/NI	-
**Missing outcome data**	●○○ Low risk of bias	3.1	Y/PY/PN/N/NI	Information specified in the results tables, where all participating subjects appear.
		3.2	NA/Y/PY/PN/N	-
		3.3	NA/Y/PY/PN/N/NI	-
		3.4	NA/Y/PY/PN/N/NI	-
**Measurement of the outcome**	●○○ Low risk of bias	4.1	PN/N	"Measurement and assessment were done at the beginning of the program (pre-test) and measurement and assessment (post-test) were done after the end of the treatment program."
		4.2	PN/N	The document and the tables of results demonstrate that the measurements were carried out in the same manner.
		4.3	Y/PY	"The study involved 16 children diagnosed with cerebral palsy (GMFCS 1-2). Selection criteria were diagnosed children with CP, GMFCS level 1-2"
		4.4	PN/N	Nothing indicates an influence on the intervention due to knowledge of the results.
		4.5	NA/Y/PY/PN/N/NI	-
**Selection of the reported results**	●○○ Low risk of bias	5.1	Y/PY/PN/N/NI	All results are shown in detail.
		5.2	PN/N	"assessment tools Berg, GMFM, and footprint plate."
		5.3	PN/N	"Measurement and assessment were done at the beginning of the program (pre-test) and measurement and assessment (post-test) were done after the end of the treatment program."
**OVERALL***	●●○ Some concerns		●●● High risk of bias	The study is judged to be at high risk of bias in at least one domain for this result OR the study is judged to have some concerns for multiple domains in a way that substantially lowers confidence in the result.
			●●○ Some concerns	The study is judged to raise some concerns in at least one domain for this result, but not to be at high risk of bias for any domain

			●○○<br>Low risk of bias	The study is judged to be at low risk of bias for all domains for this result.

*Reference: Sterne, J. A., Savović, J., Page, M. J., Elbers, R. G., Blencowe, N. S., Boutron, I., & Higgins, J. P. (2019). RoB 2: a revised tool for assessing risk of bias in randomised trials. *bmj*, *366*.

Reference: DIMITRIJEVIĆ ET AL - 2012

Variable assessed: xxxx
Page: xxxx **Figure/Table:** xxxx
Assessor name: Francisco Javier Cánovas Hernández
Date: 31/10/2025

Summary Table

Study reference	Study design	Domain 1	Domain 2	Domain 3	Domain 4	Domain 5	Overall
DIMITRIJEVIĆ ET AL - 2012	RCT	●●○ Some concerns	●○○ Low risk of bias	●○○ Low risk of bias	●○○ Low risk of bias	●●○ Some concerns	●●○ Some concerns

●●●High risk of bias, ●●○ Some concerns, ●○○Low risk of bias

Domain 1: Random sequence generation; **Domain 2:** Deviations from the intended intervention; **Domain 3:** Missing outcome data; **Domain 4:** Measurement of the outcome; **Domain 5:** Selection of reported result

Assessment Table

N tot	N int	N con	Time int	Participants	Interventions	Comparator	Outcome	Finding
xxxx	xxxx	xxxx	xxxx	xxxx	xxxx	xxxx	xxxx	xxxx

Outcome variable	Device	Partial Reliability	Absolute Reliability	Other procedures characteristics
xxxx	xxxx	xxxx	xxxx	xxxx

Study characteristics

Bias Domain	Judgement	Q		Support for judgement
Random sequence generation	●●○ Some concerns	1.1	Y/PY	"were randomly divided in two groups."
		1.2	Y/PY	"were randomly divided in two groups."
		1.3	Y/PY	"Fourteen children completed the aquatic intervention (EG), and 13 children completed all measurements but only participated as the no-intervention/control group (CG)."
Deviations from the intended intervention	●○○ Low risk of bias	2.1	Y/PY	"All parents and children had the procedures explained to them and were asked to provide informed consent prior to data collection."
		2.2	Y/PY	"The main investigator performed the aquatic therapy with the assistance of 3 additional instructors."
		2.3	PN/N	"The main objective of the swimming program was to improve safety and functional independence in the water. Each participant was taught by one instructor."
		2.4	NA/Y/PY/PN/N/NI	-
		2.5	NA/Y/PY/PN/N/NI	-
		2.6	Y/PY	"Statistical processing of all parameters was performed by calculating the mean values and standard deviation, while statistical significance ($p < 0.05$) was determined by Student's t-tests. Statistical analysis was performed with SAS version 9.1.3."
		2.7	NA/Y/PY/PN/N/NI	-
Missing outcome data	●○○ Low risk of bias	3.1	PN/N	"After starting the intervention, 2 children had to stop due to illness"
		3.2	Y/PY	Yes, they explain that those two subjects were unable to even begin the tests.
		3.3	NA/Y/PY/PN/N/NI	-
		3.4	NA/Y/PY/PN/N/NI	-
Measurement of the outcome	●○○ Low risk of bias	4.1	PN/N	"The primary outcome measures were the Gross Motor Function Measure 88 (GMFM-88) for motor function and the Water Orientation Test Alyn 2 (WOTA2) for aquatic skills assessment."
		4.2	PN/N	"…were recorded for all study participants."
		4.3	Y/PY	Clearly indicates that the examiners were aware of the outcomes.
		4.4	PN/N	"Statistical processing of all parameters was performed by calculating the mean values and standard deviation, while statistical significance ($p < 0.05$) was determined by Student's t-tests. Statistical analysis was performed with SAS version 9.1.3."
		4.5	NA/Y/PY/PN/N/NI	-
Selection of the reported results	●●○ Some concerns	5.1	Y/PY/PN/N/NI	-
		5.2	NI	"The following measures are calculated: mental adaptation (WMA), skills balance control movement (WSBM) and total score (WTOT)."

		5.3	PN/N	"All measurement were repeated at the beginning and end of intervention and after 3 weeks of follow-up after cessation of intervention"
**OVERALL***	●●○ Some concerns		●●● High risk of bias	The study is judged to be at high risk of bias in at least one domain for this result OR the study is judged to have some concerns for multiple domains in a way that substantially lowers confidence in the result
			●●○ Some concerns	The study is judged to raise some concerns in at least one domain for this result, but not to be at high risk of bias for any domain
			●○○ Low risk of bias	The study is judged to be at low risk of bias for all domains for this result.

*Reference: Sterne, J. A., Savović, J., Page, M. J., Elbers, R. G., Blencowe, N. S., Boutron, I., & Higgins, J. P. (2019). RoB 2: a revised tool for assessing risk of bias in randomised trials. *bmj*, *366*.

Reference: KHAN & IBRAHIM - 2025

Variable assessed: xxxx
Page: xxxx **Figure/Table:** xxxx
Assessor name: Francisco Javier Cánovas Hernández
Date: 31/10/2025

Summary Table

Study reference	Study design	Domain 1	Domain 2	Domain 3	Domain 4	Domain 5	Overall
KHAN & IBRAHIM - 2025	RCT	●●○ Some concerns	●○○ Low risk of bias	●○○ Low risk of bias	●●○ Some concerns	●●● High risk of bias	●●● High risk of bias

●●●High risk of bias, ●●○ Some concerns, ●○○Low risk of bias

Domain 1: Random sequence generation; **Domain 2:** Deviations from the intended intervention; **Domain 3:** Missing outcome data; **Domain 4:** Measurement of the outcome; **Domain 5:** Selection of reported result

Assessment Table

N tot	N int	N con	Time int	Participants	Interventions	Comparator	Outcome	Finding
xxxx	xxxx	xxxx	xxxx	xxxx	xxxx	xxxx	xxxx	xxxx

Outcome variable	Device	Partial Reliability	Absolute Reliability	Other procedures characteristics
xxxx	xxxx	xxxx	xxxx	xxxx

Study characteristics

Bias Domain	Judgement	Q		Support for judgement
**Random sequence generation**	●●○ Some concerns	1.1	Y/PY/PN/N/NI	-
		1.2	NI	"This study employed a randomized, unblended parallel study conducted in 2017 and 2018. A total of 48 children diagnosed with spastic cerebral palsy were evenly distributed into three matching groups (16 children per group)."
		1.3	PN/N	"The participants in the group were randomly assigned to one of the three treatment interventions: Swimming (SW), aquatic strengthening (AS), or aquatic play (AP)."
**Deviations from the intended intervention**	●○○ Low risk of bias	2.1	Y/PY	"…unblended parallel study…"
		2.2	Y/PY	"…unblended parallel study…"
		2.3	PN/N	"The participants in the group were randomly assigned to one of the three treatment interventions"
		2.4	NA/Y/PY/PN/N/NI	-
		2.5	NA/Y/PY/PN/N/NI	-
		2.6	Y/PY	"it employed functional performance outcomes comparable to the current research, such as endurance and muscle power, making it a reliable basis for sample size estimation."
		2.7	NA/Y/PY/PN/N/NI	-
**Missing outcome data**	●○○ Low risk of bias	3.1	PN/N	"Ultimately, 48 children were included in the study, with 39 children…completing all study procedures being included in the final análisis"
		3.2	Y/PY	"All participants in the SW, AS, and AP groups underwent Assessments before treatment (baseline) and after an 8-week treatment period."
		3.3	NA/Y/PY/PN/N/NI	-
		3.4	NA/Y/PY/PN/N/NI	-
**Measurement of the outcome**	●●○ Some concerns	4.1	PN/N	"The assessments covered demographics, anthropometrics, clinical characteristics, muscle tone, muscle power, balance, endurance, and functional independence and skills."
		4.2	PN/N	"All participants in the SW, AS, and AP groups underwent Assessments before treatment (baseline) and after an 8-week treatment period."
		4.3	Y/PY	The evaluators are aware of the results and improvements on a daily basis, as they are present during treatment.
		4.4	NA/Y/PY	It is likely that this knowledge may have influenced the intervention, according to the study.
		4.5	PN/N	"The collected data was entered into an Excel spreadsheet, and Statistical Package for Social Sciences (SPSS version 16.00; SPSS Inc., Chicago) was used for data analyses." "The following statistical techniques were used for each outcome"
**Selection of the reported results**	●●● High risk of bias	5.1	Y/PY/PN/N/NI	-
		5.2	Y/PY	Multiple variables are measured.
		5.3	Y/PY/PN/N/NI	-
**OVERALL***	●●● High risk of bias		●●● High risk of bias	The study is judged to be at high risk of bias in at least one domain for this result OR the study is judged to have some concerns for multiple domains in a way that substantially lowers confidence in the result.

		●●○<br>Some concerns	The study is judged to raise some concerns in at least one domain for this result, but not to be at high risk of bias for any domain
		●○○<br>Low risk of bias	The study is judged to be at low risk of bias for all domains for this result.

*Reference: Sterne, J. A., Savović, J., Page, M. J., Elbers, R. G., Blencowe, N. S., Boutron, I., & Higgins, J. P. (2019). RoB 2: a revised tool for assessing risk of bias in randomised trials. *bmj*, *366*.

Reference: NACZK ET AL - 2021

Variable assessed: xxxx
Page: xxxx **Figure/Table:** xxxx
Assessor name: Francisco Javier Cánovas Hernández
Date: 31/10/2025

Summary Table

Study reference	Study design	Domain 1	Domain 2	Domain 3	Domain 4	Domain 5	Overall
NACZK ET AL - 2021	RCT	●●○ Some concerns	●○○ Low risk of bias	●○○ Low risk of bias	●○○ Low risk of bias	●●● High risk of bias	●●● High risk of bias

●●●High risk of bias, ●●○ Some concerns, ●○○Low risk of bias

Domain 1: Random sequence generation; **Domain 2:** Deviations from the intended intervention; **Domain 3:** Missing outcome data; **Domain 4:** Measurement of the outcome; **Domain 5:** Selection of reported result

Assessment Table

N tot	N int	N con	Time int	Participants	Interventions	Comparator	Outcome	Finding
xxxx	xxxx	xxxx	xxxx	xxxx	xxxx	xxxx	xxxx	xxxx

Outcome variable	Device	Partial Reliability	Absolute Reliability	Other procedures characteristics
xxxx	xxxx	xxxx	xxxx	xxxx

Study characteristics

Bias Domain	Judgement	Q		Support for judgement
**Random sequence generation**	●●○ Some concerns	1.1	Y/PY/PN/N/NI	-
		1.2	NI	It is not clear in the paper.
		1.3	PN/N	"The participants were randomly allocated into two groups: the training group (T; n = 11; age, 14.9 _ 2.35 years; body mass, 56.8 _ 7.97 kg; height, 152 _ 8.65 cm) and the control group (C; n = 11; age, 14.4 _ 1.97 years; body mass, 57.2 _ 8.43 kg; height, 150 _ 12.0 cm) using the chit method."
**Deviations from the intended intervention**	●○○ Low risk of bias	2.1	Y/PY	"The participants (by means of a formal request communicated to their parents) were asked to maintain a normal state of hydration prior to measurement, and they were not allowed to exercise, or eat for 12 h preceding the measurements."
		2.2	Y/PY	"Attendance at all sessions was strictly monitored." They imply that investigators are aware of the treatment being received by those under investigation.
		2.3	PN/N	"…using the chit method. Each group included seven boys and four girls."
		2.4	NA/Y/PY/PN/N/NI	-
		2.5	NA/Y/PY/PN/N/NI	-
		2.6	Y/PY	The analysis carried out is correct for estimating the effects.
		2.7	NA/Y/PY/PN/N/NI	-
**Missing outcome data**	●○○ Low risk of bias	3.1	Y/PY	"The details are presented in the participants' flow chart." Results are obtained from all participants.
		3.2	NA/Y/PY/PN/N	-
		3.3	NA/Y/PY/PN/N/NI	-
		3.4	NA/Y/PY/PN/N/NI	-
**Measurement of the outcome**	●○○ Low risk of bias	4.1	PN/N	As detailed in Table 3, the measurements are adequate for the parameters sought.
		4.2	PN/N	The measurements were taken in exactly the same way in both groups.
		4.3	Y/PY	The examiners knew the results of the intervention, as understood from the study.
		4.4	PN/N	"To evaluate the influence of 33 weeks of exercise on body composition, bioelectrical impedance device (Tanita MC-980 MA, Tanita Corporation, Tokyo, Japan) was used. The participants (by means of a formal request communicated to their parents) were asked to maintain a normal state of hydration prior to measurement, and they were not allowed to exercise, or eat for 12 h preceding the measurements. The measurements were made in the morning, according to manufacturer's guidelines." When carrying out this type of measurement, there is no possibility of an error of influence in the intervention.
		4.5	NA/Y/PY/PN/N/NI	-
	●●● High risk of bias	5.1	Y/PY/PN/N/NI	-

**Selection of the reported results**		5.2	Y/PY	Several outcomes were evaluated, as shown in Table 3.
		5.3	Y/PY/PN/N/NI	-
**OVERALL***	●●● High risk of bias		●●● High risk of bias	The study is judged to be at high risk of bias in at least one domain for this result OR the study is judged to have some concerns for multiple domains in a way that substantially lowers confidence in the result.
			●●○ Some concerns	The study is judged to raise some concerns in at least one domain for this result, but not to be at high risk of bias for any domain
			●○○ Low risk of bias	The study is judged to be at low risk of bias for all domains for this result.

*Reference: Sterne, J. A., Savović, J., Page, M. J., Elbers, R. G., Blencowe, N. S., Boutron, I., & Higgins, J. P. (2019). RoB 2: a revised tool for assessing risk of bias in randomised trials. *bmj*, *366*.

Reference: SALARI ET AL -2025

Variable assessed: xxxx
Page: xxxx **Figure/Table:** xxxx
Assessor name: Francisco Javier Cánovas Hernández
Date: 31/10/2025

Summary Table

Study reference	Study design	Domain 1	Domain 2	Domain 3	Domain 4	Domain 5	Overall
SALARI ET AL - 2025	RCT	●○○ Low risk of bias	●○○ Low risk of bias	●○○ Low risk of bias	●○○ Low risk of bias	●○○ Low risk of bias	●○○ Low risk of bias

●●●High risk of bias, ●●○ Some concerns, ●○○Low risk of bias

Domain 1: Random sequence generation; **Domain 2:** Deviations from the intended intervention; **Domain 3:** Missing outcome data; **Domain 4:** Measurement of the outcome; **Domain 5:** Selection of reported result

Assessment Table

N tot	N int	N con	Time int	Participants	Interventions	Comparator	Outcome	Finding
xxxx	xxxx	xxxx	xxxx	xxxx	xxxx	xxxx	xxxx	xxxx

Outcome variable	Device	Partial Reliability	Absolute Reliability	Other procedures characteristics
xxxx	xxxx	xxxx	xxxx	xxxx

Study characteristics

Bias Domain	Judgement	Q		Support for judgement
**Random sequence generation**	●○○ Low risk of bias	1.1	Y/PY	"For the present study, a single-blind Randomized Controlled Trial (RCT) design was employed following CONSORT reporting guidelines"
		1.2	Y/PY	"For the present study, a single-blind Randomized Controlled Trial (RCT) design was employed following CONSORT reporting guidelines"
		1.3	PN/N	"Finally, the results of 15 participants in the CO group and 15 participants in the EX group were included in the final analysis."
**Deviations from the intended intervention**	●○○ Low risk of bias	2.1	Y/PY	"participants were individually acquainted with the experimental tests and procedures"
		2.2	Y/PY	"During this session, researchers provided detailed explanations of each test and addressed any questions or concerns raised by the participants"
		2.3	PN/N	"This approach was designed to enhance participant understanding and comfort with the procedures, reducing any anxiety or confusion about new testing methods. Special attention was given to standardizing the testing procedures across all participants, such as ensuring consistent positioning of equipment, maintaining the same time duration for each test, following identical instructions for every participant, standardized lighting, temperature and noise levels to minimize potential biases or variations in test administration."
		2.4	NA/Y/PY/PN/N/NI	-
		2.5	NA/Y/PY/PN/N/NI	-
		2.6	Y/PY	"Careful attention was paid to positioning the electrodes over the target muscles of interest, including the tibialis anterior (TA), gastrocnemius medialis (GM), rectus femoris (RF), and biceps femoris (BF)."
		2.7	NA/Y/PY/PN/N/NI	
**Missing outcome data**	●○○ Low risk of bias	3.1	Y/PY	"During the 8-week intervention period, no participants withdrew from the study." And the evaluation table indicates that all were measured.
		3.2	NA/Y/PY/PN/N	-
		3.3	NA/Y/PY/PN/N/NI	-
		3.4	NA/Y/PY/PN/N/NI	-
**Measurement of the outcome**	●○○ Low risk of bias	4.1	Y/PY/PN/N/NI	"These electrodes, consisting of disposable silver/silver chloride (AgCl-Ag), were placed on the skin according to the recommendations outlined in the Surface Electromyography for the Non-Invasive Assessment of Muscles (SENIAM) guidelines"
		4.2	Y/PY/PN/N/NI	All participants were measured and results were obtained for all of them.
		4.3	PN/N	"However, to reduce potential biases, a researcher conducted random weekly check-ins with control group members to monitor their activities and general well-being throughout the study."
		4.4	NA/Y/PY/PN/N/NI	-
		4.5	NA/Y/PY/PN/N/NI	-
**Selection of the reported results**	●○○ Low risk of bias	5.1	Y/PY/PN/N/NI	All results obtained are detailed.
		5.2	Y/PY/PN/N/NI	"Muscle strength was quantified using the Lafayette Handheld Dynamometer, with specific protocols and procedures tailored to each muscle group of interest"

		5.3	Y/PY/PN/N/NI	"If a significant interaction was found between factors, post hoc paired t-tests with Bonferroni correction for multiple comparisons were applied."
**OVERALL***	●○○ Low risk of bias		●●● High risk of bias	The study is judged to be at high risk of bias in at least one domain for this result OR the study is judged to have some concerns for multiple domains in a way that substantially lowers confidence in the result.
			●●○ Some concerns	The study is judged to raise some concerns in at least one domain for this result, but not to be at high risk of bias for any domain
			●○○ Low risk of bias	The study is judged to be at low risk of bias for all domains for this result.

*Reference: Sterne, J. A., Savović, J., Page, M. J., Elbers, R. G., Blencowe, N. S., Boutron, I., & Higgins, J. P. (2019). RoB 2: a revised tool for assessing risk of bias in randomised trials. *bmj*, *366*.

Reference: ZHAO ET AL - 2024

Variable assessed: xxxx
Page: xxxx **Figure/Table:** xxxx
Assessor name: Francisco Javier Cánovas Hernández
Date: 31/10/2025

Summary Table

Study reference	Study design	Domain 1	Domain 2	Domain 3	Domain 4	Domain 5	Overall
ZHAO ET AL - 2024	RCT	●○○ Low risk of bias	●○○ Low risk of bias	●○○ Low risk of bias	●○○ Low risk of bias	●○○ Low risk of bias	●○○ Low risk of bias

●●●High risk of bias, ●●○ Some concerns, ●○○Low risk of bias

Domain 1: Random sequence generation; **Domain 2:** Deviations from the intended intervention; **Domain 3:** Missing outcome data; **Domain 4:** Measurement of the outcome; **Domain 5:** Selection of reported result

Assessment Table

N tot	N int	N con	Time int	Participants	Interventions	Comparator	Outcome	Finding
xxxx	xxxx	xxxx	xxxx	xxxx	xxxx	xxxx	xxxx	xxxx

Outcome variable	Device	Partial Reliability	Absolute Reliability	Other procedures characteristics
xxxx	xxxx	xxxx	xxxx	xxxx

Study characteristics

Bias Domain	Judgement	Q		Support for judgement
**Random sequence generation**	●○○ Low risk of bias	1.1	Y/PY	"Participants were randomly assigned to the aquatic exercise group, floor curling group, or control group."
		1.2	Y/PY	"This study employed a randomized controlled trial design, with randomization performed using computer-generated randomization."
		1.3	PN/N	"with an expected allocation ratio of 1:1:1. Ultimately, 14 participants were assigned to the aquatic exercise group (age = 10.79 } 1.05, IQ = 39.21 } 11.98, height = 1.45 } 0.05 m, weight = 40.51 } 6.30 kg), 14 participants were assigned to the floor curling group (age = 10.50 } 1.02, IQ = 38.21 } 13.73, height = 1.42 } 0.05 m, weight = 39.81 } 5.33 kg), and 14 participants were assigned to the control group (age = 10.43 } 1.10, IQ = 40.14 } 13.24, height = 1.42 } 0.07 m, weight = 40.27 } 5.49 kg)."
**Deviations from the intended intervention**	●○○ Low risk of bias	2.1	Y/PY	"possessing basic language comprehension skills to cooperate with the researchers, (c) able to participate in the intervention programs and testing tasks within a designated time with the accompaniment of guardians"
		2.2	Y/PY	"This study was conducted at a local disability training center with which our research team has established a long-term collaborative relationship." "The research team provided a thorough explanation of this study's purpose and procedures to the participants' caregivers, sought input from the children, and obtained parental informed consent before collecting the information."
		2.3	PN/N	Does not affect randomisation. "This was carried out to minimize any potential confounding factors related to balance ability test performance. The PAQ-C has demonstrated good test–retest reliability (R = 0.82)"
		2.4	NA/Y/PY/PN/N/NI	-
		2.5	NA/Y/PY/PN/N/NI	-
		2.6	Y/PY	The analysis is adequate for estimating the desired effects.
		2.7	NA/Y/PY/PN/N/NI	-
**Missing outcome data**	●○○ Low risk of bias	3.1	Y/PY	"each comprising 14 children with ID who participated in the entire intervention and testing process."
		3.2	NA/Y/PY/PN/N	-
		3.3	NA/Y/PY/PN/N/NI	-
		3.4	NA/Y/PY/PN/N/NI	-
**Measurement of the outcome**	●○○ Low risk of bias	4.1	PN/N	"The PAQ-C has demonstrated good test–retest reliability (R = 0.82)"
		4.2	PN/N	"each comprising 14 children with ID who participated in the entire intervention and testing process." They indicate that all groups were measured in a similar manner.
		4.3	Y/PY	"The collected demographic information included participants' gender, age, height, weight,

				intelligence quotient level, and other relevant basic details.”
		4.4	PN/N	“This was carried out to minimize any potential confounding factors related to balance ability test performance.”
		4.5	NA/Y/PY/PN/N/NI	-
**Selection of the reported results**	●○○ Low risk of bias	5.1	Y/PY	“As shown in Table 3, there were no significant differences between the three groups of participants in terms of age, body mass index (BMI), physical activity, intelligence quotient (IQ), or other variables at baseline (pre-test). Additionally, no statistically significant differences were found among the three groups in the Berg Balance Test and lower limb muscle strength measures.”
		5.2	PN/N	“In this study, the Berg Balance Test was employed to assess participants’ balance ability.” “In this study, the participants’ lower limb muscle strength was assessed using a handheld dynamometer (HHD) known as the HogganR MicroFET3.”
		5.3	PN/N	“The normality and homogeneity of variance were confirmed using the Shapiro–Wilk test and Levene’s test, respectively. Continuous variables following a normal distribution were presented as means (M) } standard deviations (SD).”
**OVERALL***	●○○ Low risk of bias		●●● High risk of bias	The study is judged to be at high risk of bias in at least one domain for this result OR the study is judged to have some concerns for multiple domains in a way that substantially lowers confidence in the result.
			●●○ Some concerns	The study is judged to raise some concerns in at least one domain for this result, but not to be at high risk of bias for any domain
			●○○ Low risk of bias	The study is judged to be at low risk of bias for all domains for this result.

*Reference: Sterne, J. A., Savović, J., Page, M. J., Elbers, R. G., Blencowe, N. S., Boutron, I., & Higgins, J. P. (2019). RoB 2: a revised tool for assessing risk of bias in randomised trials. *bmj*, *366*.
